# Use of deep learning methods to translate drug-induced gene expression changes from rat to human primary hepatocytes

**DOI:** 10.1371/journal.pone.0236392

**Published:** 2020-08-11

**Authors:** Shauna D. O’Donovan, Kurt Driessens, Daniel Lopatta, Florian Wimmenauer, Alexander Lukas, Jelmer Neeven, Tobias Stumm, Evgueni Smirnov, Michael Lenz, Gokhan Ertaylan, Danyel G. J. Jennen, Natal A. W. van Riel, Rachel Cavill, Ralf L. M. Peeters, Theo M. C. M. de Kok

**Affiliations:** 1 Maastricht Centre for Systems Biology (MaCSBio), Maastricht University, Maastricht, The Netherlands; 2 Division of Human Nutrition and Health, Wageningen University and Research, Wageningen, The Netherlands; 3 Dept. of Data Science and Knowledge Engineering, Maastricht University, Maastricht, The Netherlands; 4 Institute of Organismic and Molecular Evolution, Johannes Gutenberg University Mainz, Mainz, Germany; 5 Preventive Cardiology and Preventative Medicine—Center for Cardiology, University Medical Center of the Johannes Gutenberg University Mainz, Mainz, Germany; 6 Flemish Institute for Technological Research (VITO), Mol, Belgium; 7 Dept. of Toxicogenomics, GROW School for Oncology and Developmental Biology, Maastricht University, Maastricht, The Netherlands; 8 Dept. of Biomedical Engineering, Eindhoven University of Technology, The Netherlands; National University of Sciences and Technology, PAKISTAN

## Abstract

In clinical trials, animal and cell line models are often used to evaluate the potential toxic effects of a novel compound or candidate drug before progressing to human trials. However, relating the results of animal and *in vitro* model exposures to relevant clinical outcomes in the human *in vivo* system still proves challenging, relying on often putative orthologs. In recent years, multiple studies have demonstrated that the repeated dose rodent bioassay, the current gold standard in the field, lacks sufficient sensitivity and specificity in predicting toxic effects of pharmaceuticals in humans. In this study, we evaluate the potential of deep learning techniques to translate the pattern of gene expression measured following an exposure in rodents to humans, circumventing the current reliance on orthologs, and also from *in vitro* to *in vivo* experimental designs. Of the applied deep learning architectures applied in this study the convolutional neural network (CNN) and a deep artificial neural network with bottleneck architecture significantly outperform classical machine learning techniques in predicting the time series of gene expression in primary human hepatocytes given a measured time series of gene expression from primary rat hepatocytes following exposure *in vitro* to a previously unseen compound across multiple toxicologically relevant gene sets. With a reduction in average mean absolute error across 76 genes that have been shown to be predictive for identifying carcinogenicity from 0.0172 for a random regression forest to 0.0166 for the CNN model (p < 0.05). These deep learning architecture also perform well when applied to predict time series of *in vivo* gene expression given measured time series of *in vitro* gene expression for rats.

## Introduction

The field of toxicology primarily concerns itself with the evaluation of compounds for potential adverse effects in humans. The liver is the primary organ for metabolism and detoxification of pharmaceuticals and other compounds, consequently adverse effects most often occur in the liver. However, the liver is not readily accessible for sampling. In the absence of relevant human data a plethora of alternative bioassays are used to predict adverse toxicological outcomes in humans. The current ‘gold standard’ for evaluating the toxicity of a compound is the two-year rodent bioassay [1]. However, this method is time-consuming, expensive, and requires the sacrifice of large numbers of animals in order to screen a single compound. Moreover, the predictive relevance of the rodent bioassay is increasingly being called into question, with a 2009 report by Hartung indicating that tests in rodents had correctly predicted just 43% of toxic effects of pharmaceuticals in humans [2]. Given the high attrition rates of novel candidate drugs in the final stages of research and drug development having alternatives to animal toxicity tests that are more reliable is of particular concern to the pharmaceutical industry [2]. Coupled with the increasingly restrictive legislation surrounding animal testing, this has motivated the development of alternatives, mostly based on human *in vitro* cellular models.

The intra-cellular response to an external stimulus, such as a potentially toxic exposure, is mediated by gene transcripts, also known as mRNA. The gene transcripts relay the genomic information stored within our DNA in the nucleus of each cell allowing proteins necessary to metabolise and detoxify the compound to be translated and activated, in accordance with the central dogma of molecular biology. With advancements in high-throughput omics platforms such as micro-array, and more recently RNA-Seq, it is now possible to measure the mRNA levels within a sample. Such transcriptomic profiles not only provide a particularly sensitive assessment of the cellular response to a potentially toxic external stimulus, but as they capture genome-wide alteration in mRNA levels following an exposure they may also facilitate mechanistic insight into modes of toxicological action or disease development.*in vitro* toxicogenomic assays have produced promising results in the discrimination between subclasses of carcinogenicity [3, 4] with an overall accuracy of 80% for the prediction of *in vivo* rodent toxicity [5–10]. Nevertheless, *in vitro* assays are not without their limitations. These simple cell systems differ greatly in terms of functionality and signalling from the organs they represent; lacking the systemic interplay with other tissues that exists *in vivo*. This is more so a problem with commonly used immortalized cell lines, such as tumour derived hepatocytes HepG2, where liver metabolic functions tend to vanish as culture time increases [11, 12].

In addition, the analysis and interpretation of the high dimensional, highly correlated gene expression data presents many new challenges. While, multiple studies have reported positive results in predicting toxicity of a novel compound using genomic signatures of disease states [13–15], relating the genetic signatures identified from human disease states to *in vitro* or *in vivo* rodent toxicogenomics assays is achieved using, often putative, orthologs. Orthologs are genes in different species that are share a similar function or role, often predicted based on similarities in genetic sequences. However, a study which compared the toxicogenomics responses for various compounds following 24 hour exposure for HepG2 cells, primary human, rat, and murine hepatocytes noted that while the biological replicates for a given compound and model were close together no single compound induced similar responses in all *in vitro* models [16]. The discrepancy between gene expression patterns between orthologs in different cellular models highlights the need for methods that can help us bridge not only the cross-species division but also resolve differences resulting from experimental techniques. Allowing for extrapolation of functional and mechanistic changes in cellular responses to the human *in vivo* disease state.

Artificial Neural Networks (ANNs) have emerged as a highly successful field of machine learning. ANNs consist of several layers of forward connected nodes, which transform the raw data into increasingly abstract feature representations for use in classification or prediction [17–19]. While neural networks themselves are not a novel idea, advances in computational power and training algorithms in more recent years, coupled with the availability of sufficiently large data sets, have allowed deeper architectures to be trained [20]. Deep ANNs have produced outstanding results on a range of applications including natural language [21] and image processing [22] and have been mooted as being a potential solution to the challenges faced with analysing high dimensional toxicogenmics data [23]. Deep learning methods have already been shown to outperform more classical techniques in predicting toxicity [24, 25] and drug-induced liver injury [26, 27] given information about the chemical structure and properties of a compound. In addition, multiple recent studies have had some success in applying deep learning architectures to the analysis of gene expression data, predicting genome-wide gene expression given information on histone modifications [28] or expression levels for a subset of, so-called, landmark genes [29]. Within the field of toxicology, deep learning models have also been successfully applied to predict the pharmacological properties of drugs given transcriptomic profiles, facilitating drug repurposing [30] and also predicting liver toxicity [31].

In this study, we evaluate the potential of deep learning methods to extrapolate transcriptomic profiles from rodent to human and from *in vitro* to *in vivo* in rats. Several deep learning network architectures are compared with more classical machine learning approaches, such as random regression forests and k-nearest neighbours, to determine the added value of the deep learning approaches. Firstly, we examine the cross-species prediction problem, applying the models to predict human *in vitro* gene expression given a time series of rat *in vitro* gene expression following exposure to a previously unseen compound. Subsequently, we look at predicting rat *in vivo* gene expression given a measured pattern of rat *in vitro* gene expression following an exposure using data from open TG-GATEs. In addition, a systematic analysis is conducted to determine the effect of the composition of the included genes on the accuracy of the model predictions.

## Materials and methods

### Open TG-GATEs

**Fig 1:**
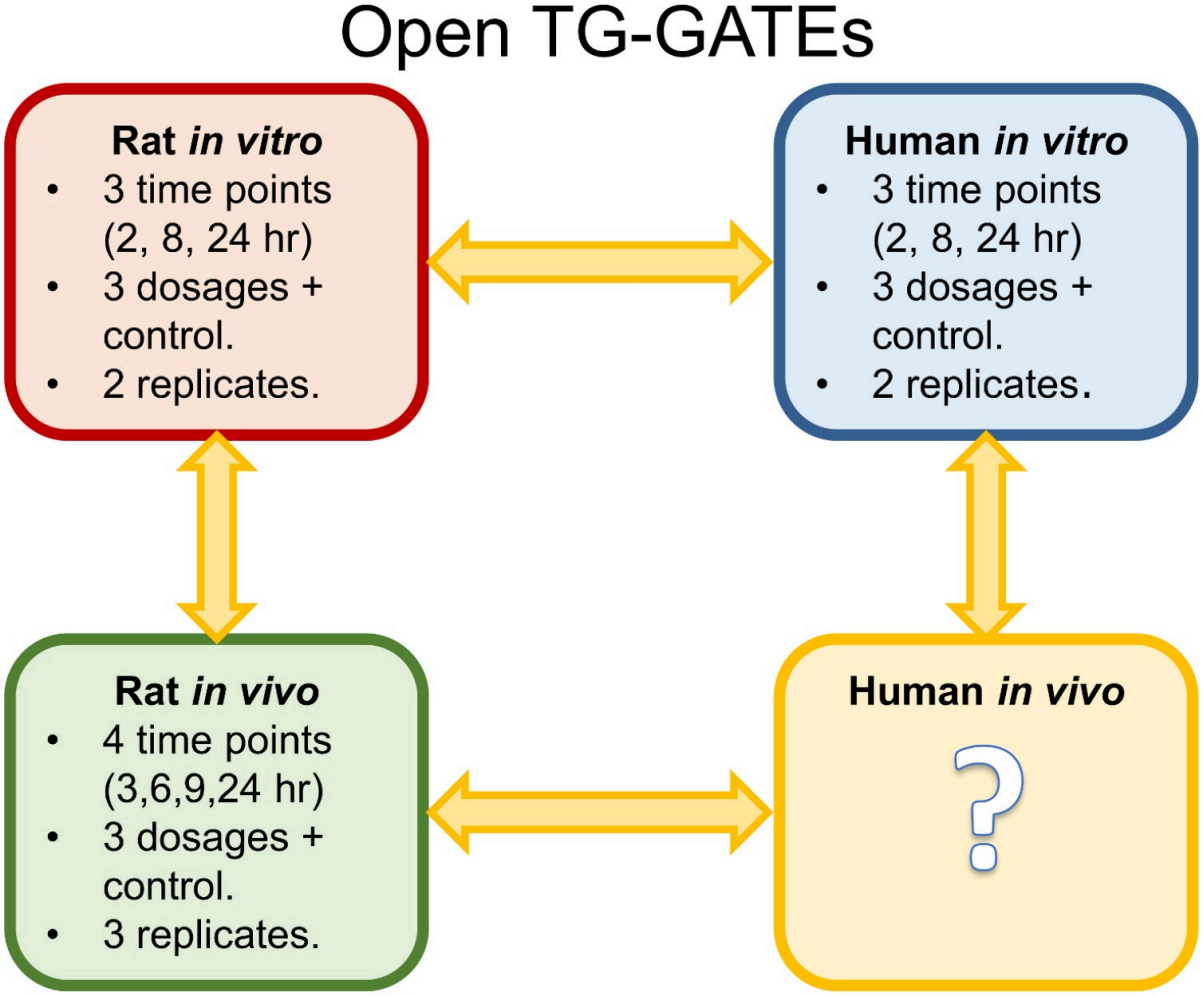
Overview of toxicogenomics data available in open TG-GATEs. Outline of toxicogenomics data available in Open TG-GATEs across three domains. In rat and human *in vitro* studies gene expression profiles are measured at three time points (2, 8, 24 hours) with two biological replicates at low, medium, and high dosages plus the control. The rat *in vivo* data is available at four time points (3, 6, 9, and 24 hours) at a low, medium, and high dosage plus a control with three biological replicates. No data is available for the human *in vivo* domain.

Open TG-GATEs is a large, publicly available toxicogenomics database containing gene expression profiles as well as traditional toxicological data from *in vitro* assays, in both primary rat and human hepatocytes, and *in vivo* rats following exposure to 170 compounds [32]. The data used in these analyses are summarised in Fig 1. For the *in vitro* human and rat experiments gene expression profiles were measured at three time points (2, 8, and 24 hours) following a single exposure to a given compound at up to three dosages (low, middle, and high) plus a control; with two biological replicates. The high dosage for each compound was determined as the maximally tolerated dose (yielding an 80-90% relative survival ratio). The ratio of concentrations for the low and middle doses were 1/25 and 1/5 of the high dose. For the rat *in vivo* experiments, gene expression profiles were measured at four time points (3, 6, 9, and 24 hours) following a single exposure. The high dosage for each compound was selected to match the level shown to induce the minimum toxic effect over the course of a four week toxicity study, with the ratio of concentrations for the low and middle doses at 1/10 and 3/10 of the high dose. There are three biological replicates for each *in vivo* compound-dosage combination. Gene expression profiles were generated using the Affymetrix GeneChip, the Rat Genome 230 2.0 Array and the Human Genome U133 Plus 2.0 Array. Array data for all available compounds for rat and human *in vitro* and rat *in vivo* were downloaded in the form of CEL files from the Open TG-GATEs database (https://toxico.nibiohn.go.jp) and pre-processed using Affymetrix Power Tools using the robust multi-array average normalisation method, correcting the raw micro-array probe intensities for background and inter-array noise to produce interpretable gene expression data. Following normalisation, compounds with incomplete data, missing either time points or dosages, were removed, leaving 45 compounds with a complete set of data in all three domains which were included in this study (Supplementary Table S1 in S1 File).

### Experimental setup

**Fig 2:**
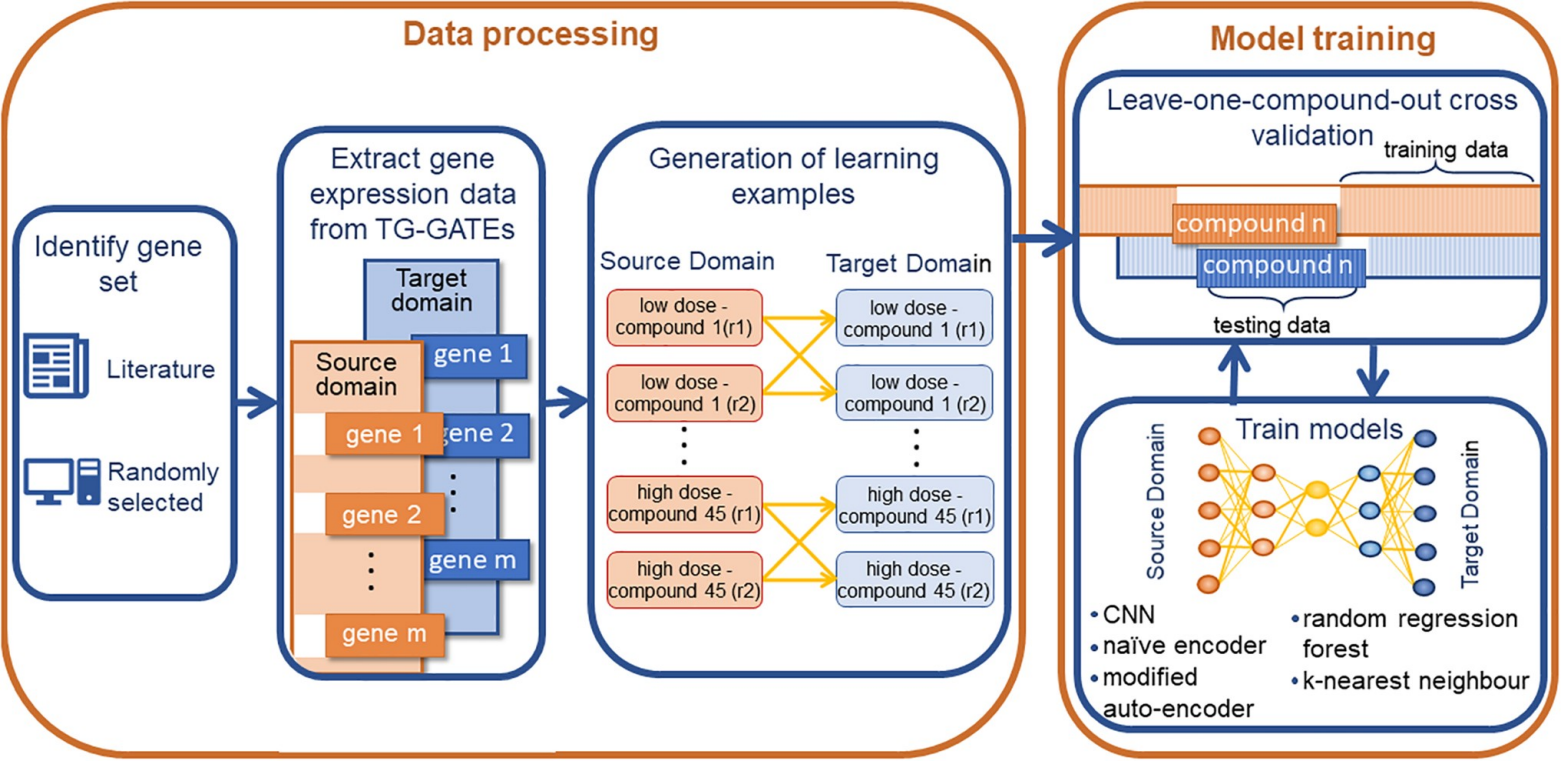
Schematic of experimental setup. Overview of the design of this study. Firstly, gene sets are identified either from literature or by randomly selecting genes from a list of known rat-human orthologs. Once the relevant gene expression data has been extracted from the TG-GATEs data set and learning examples are generated by the pairwise matching of compound dose combinations. The models are then trained using a leave-one-compound-out cross validation loop, where by all instance for one compound are removed from the data set, the models are then trained on the data for the remaining 44 compounds and predictions assessed by predicting for this unseen compound.

The experimental setup of this study is visualised in Fig 2 and detailed below. In summary, gene sets are identified either from literature or randomly selected from a list of known rat-human orthologs. Once the relevant gene expression data has been extracted from the TG-GATEs data set machine learning examples can be generated by pairwise matching of compound-dose combinations from the source (rat *in vitro*) and target (human *in vitro* or rat *in vivo*) domains. The gene expression data is then re-encoded and reformatted into the preferred input for the models. In order to maximise the number of learning examples available for training each of the models are trained using a leave-one-compound-out cross validation approach. Model performance is assessed using the average mean absolute error in predicting gene expression over all 45 compounds. A detailed description of the three applied deep learning architectures and two machine learning models can also be found below.

#### Gene sets

Micro-array data measures the abundance of more than 20,000 gene transcripts. Consequently, our time series of gene expression profiles have a very high dimensional feature space. While Open TG-GATEs is a comparatively large toxicogenomics database, the 720 learning examples that could be generated by combining all available compounds and dosages are somewhat restrictive for effectively training a model to predict genome-wide gene expression. For example, a single fully connected layer of a neural network going from n nodes in the input layer to m nodes in the internal layer requires the training of n*m weights. A genome-wide prediction for a single time-point would require an input layer consisting of 20,000 nodes resulting in 20,000*m parameters which would need to be optimised. Therefore, it was decided to select subsets of genes to evaluate the predictive ability of the neural networks. Four subsets of genes associated to relevant toxicological outcomes were identified from literature; namely steatosis (developed in-house using the KEGG pathway hsa04932), cholestasis [33–35], genotoxicity and carcinogenicity (GTX+C) [13, 14], and NAFLD [15]. The published gene lists were filtered to contain only known rat-human orthologs. Gene sets can be found in Supplementary Tables S2-S5 in S1 File.

In order to evaluate the effect of gene set composition on model predictions several randomly selected gene sets of increasing size were generated. Initially, sets of 20 genes were randomly selected independently from both the source domain (rat *in vitro*) and the target domain (human *in vitro* or rat *in vivo*). Subsequently, sets of 15 randomly selected genes were iteratively added to the core set; generating nested gene sets consisting of 35, 50, 65, and 80 genes. Random sets of orthologs were also generated by repeating this nested gene set selection procedure within a reduced set of known rat-human orthologs. The expression of some genes was not altered following the exposure. Constant gene expression is easier to predict. Therefore, all generated gene sets were screened to ensure sufficient variation in gene expression. This threshold was set at 3% lower than the variance measured in the steatosis gene set.

#### Learning examples

Once the expression data for the selected gene sets have been extracted from the TG-GATEs data set. Machine learning examples are generated by pairing the time series of gene expression for a given compound-dose combination in the source domain (rat *in vitro*) with the time series of gene expression for the same compound-dose combination in the target domain (human *in vitro* or rat *in vivo*). As each replicate for a given compound-dose combination in the source domain is a valid match for both replicates in the target domain four learning examples can be generated for each compound-dose combination (Fig 2, Supplementary Fig S1 in S1 File). In order to maintain the structure of the data in each domain one of the three replicates for the rat *in vivo* domain is randomly discarded. For the purpose of generating learning examples, the controls are treated as an additional dose. With 45 compounds, three dosages plus the control, and the pairwise matching of replicates it is possible to generate 720 learning examples for each source-target domain combination. For all models, with the exception of the convolutional neural network, gene expression data is arranged in a vector consisting of each time points of gene expression for consecutive genes for a given compound-dose combination, for example the rat *in vitro* data for the GTX+C gene set consists of gene expression values for 76 genes at three time points resulting in a (228 x 1) dimensional input vector. For the CNN the gene expression data is restructured into a two dimensional array with time points on one dimension and genes on the second, consequently the rat *in vitro* data for the GTX+C gene sets forms a (76 x 3) array.

#### Encoding the gene expression data

With time series of gene expression data, it is the changes in gene expression between consecutive time points, and not the levels of gene expression, which is of primary interest. Consequently, the input data was re-encoded, favouring the correct prediction of the pattern of gene expression. The first entry for each gene is the measured gene expression at time point one. The values have been re-scaled relative to maximum and minimum gene expression levels across all genes and compound-dose combinations such that the values are in the range [0, 1], the preferred input to neural networks. The remaining entries encode the slopes between each consecutive pair of time points. The second entry is the difference in gene expression between the first time point and the second time point and so on. This re-encoding of the gene expression data produces a lower error for predictions with the correct gene expression pattern, even if the level of gene expression has been shifted, without the need to use a more complex error function when training (Supplementary Fig S2a and S2b in S1 File).

### Leave-one-compound-out cross validation

In order to maximise the number of learning examples available for training leave-one-compound-out cross validation is used. All sixteen instances for a given compound were removed from the training data, the models were then trained on the remaining 704 learning examples. The excluded instances were then used to validate the prediction accuracy of the model. This was repeated for all 45 compounds. The overall performance of each model was assessed using the average validation error over all 45 compounds. The validation error is the mean absolute error between the model predicted time series of gene expression and the measured time series of gene expression for each gene.

Models are trained using the mean absolute error across all time points and genes according to the following formula.

$$\begin{array}{c} \text{MAE}=\frac{1}{G*T}\sum_{i=1}^{G}\sum_{j=1}^{T}\left|X_{ijc}-M_{ijc}\right| \end{array}$$ (1)

Where *T* is the number of sampled gene expression time points and *G* is the number of genes in the gene set. *X*_*ijc*_ is the measured gene expression level and *M*_*ijc*_ is the model prediction of gene expression at time point *j* for gene *i* for a given compound-dose combination *c*. Overall model performance is assessed as the average mean absolute error over the 45 leave-one-compound-out loops.

### Models

**Fig 3:**
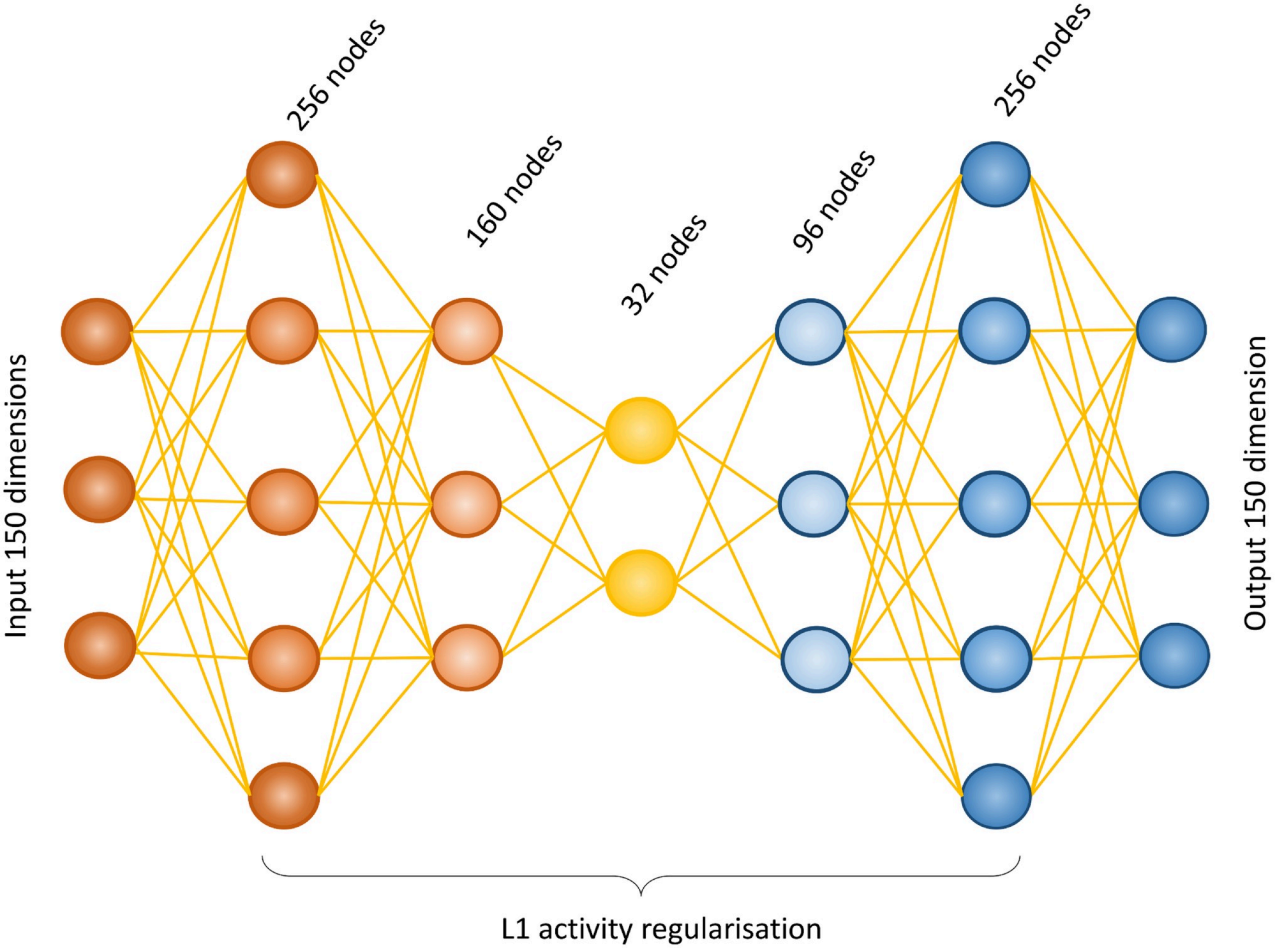
Schematic diagram of the architecture of the Naïve Encoder model. This model is a densely connected five-layer artificial neural network with a bottle neck architecture. The two encoding layers consisting of 256 and 160 nodes respectively, followed by a central bottleneck layer containing 32 nodes and two decoder layers containing 96 and 256 layers respectively. All layers use ReLU activation, other than the final output layer which uses sigmoid activation. L1 activity regulation is applied to all layers to enforce sparseness. Input and output layers are shown for the steatosis gene set in the rat *in vitro* to human *in vitro* prediction, where both the input and the output have 150 dimension (50 genes * 3 time points).

Artificial Neural Networks consist of many nodes arranged into multiple connected layers [17]. Each node processes the information received from the previous layer as a function of the inputs and their connection weights and responds accordingly. These responses, known as activations, become the input for the subsequent layer of nodes [36]. Through training the connection weights are tuned [20], prioritising the most informative features for the particular task. A bottleneck layer is a layer that contains fewer nodes compared to the previous layers, depicted in Fig 3, forcing the network to find a reduced dimensional representation of the data. The bottleneck forces learning by restricting the network to use only the most relevant features [37]. Consequently, the reduced dimensional latent space also helps avoid over-fitting, preventing learning of a one-to-one prediction. Two bottleneck architectures were implemented in this study.

#### Naive encoder

The first model is a classical, fully connected ANN with a bottleneck structure (Fig 3). Through a grid search of the hyper-parameters a well-performing network structure was found to consist of two hidden layers containing 256 and 160 nodes respectively, a central layer containing 32 nodes, followed by two further layers with 96 and 256 nodes. All layers use rectified linear unit (ReLU) activation [38], except for the output layer which uses sigmoid activation. L1 activity regularisation was applied to all five hidden layers to enforce sparseness [39]. Initial connection weights were randomly assigned.

#### Modified autoencoders

**Fig 4:**
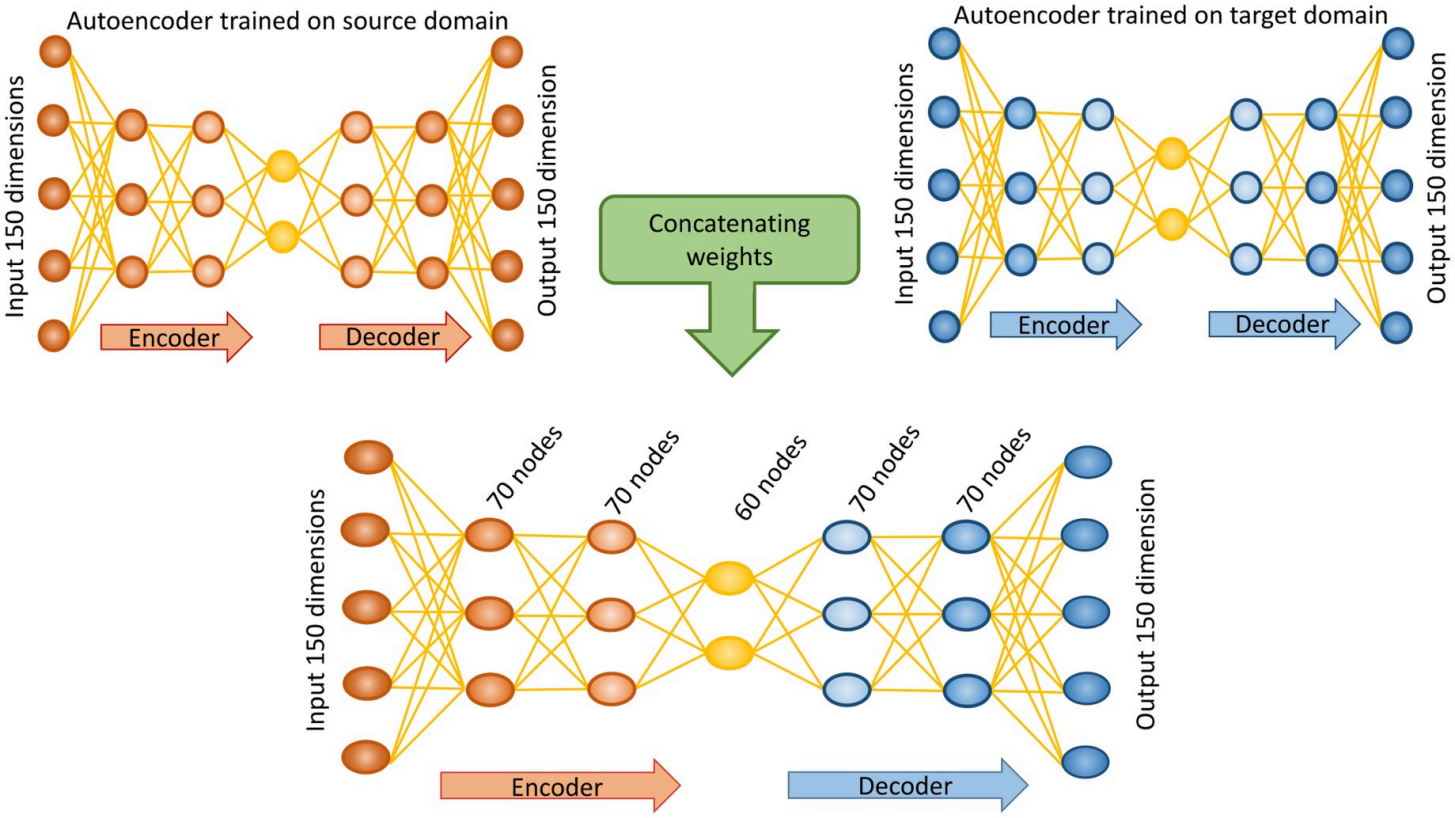
Schema for the structure of the modified autoencoder. Initially, two separate autoencoders are trained independently one for the source domain (red) and a second for the target domain (blue), these networks have the same architecture as the final modified autoencoder. The weights for the encoder portion of the source domain autoencoder (red) are concatenated with the weights for the decoder section of the target domain autoencoder (blue). These weights become the initialising weights for the prediction task. The modified autoencoder consists of five hidden layers of 70, 70, 60, 70, and 70 nodes respectively. L1 activity regularisation is applied to the three middle hidden layers. Input and output layers are shown for the steatosis gene set in the rat *in vitro* to human *in vitro* prediction, where both the input and the output have 150 dimension (50 genes * 3 time points).

Autoencoders are a subclass of ANNs with a bottleneck design, used to learn an efficient encoding of data [40]. Rather than initialising our prediction model with random weights, it was decided to independently train separate autoencoders for each of our data domains. The trained weights for the encoder for our source domain were then concatenated with the trained weights for the decoder of the target domain. These weights became the initial weights for training our second bottleneck architecture, outlined in Fig 4. This network also consists of five hidden layers. The first two layers forming the encoder contain 70 nodes, the bottleneck layer contains 60 nodes, and the remaining two layers of the decoder have 70 nodes. To stimulate the learning of compact internal representation of the data L1 activity regularisation was applied to the three middle layers of the network. All layers use ReLU activation except for the output layer, which uses sigmoid activation.

#### Convolutional neural network

**Fig 5:**
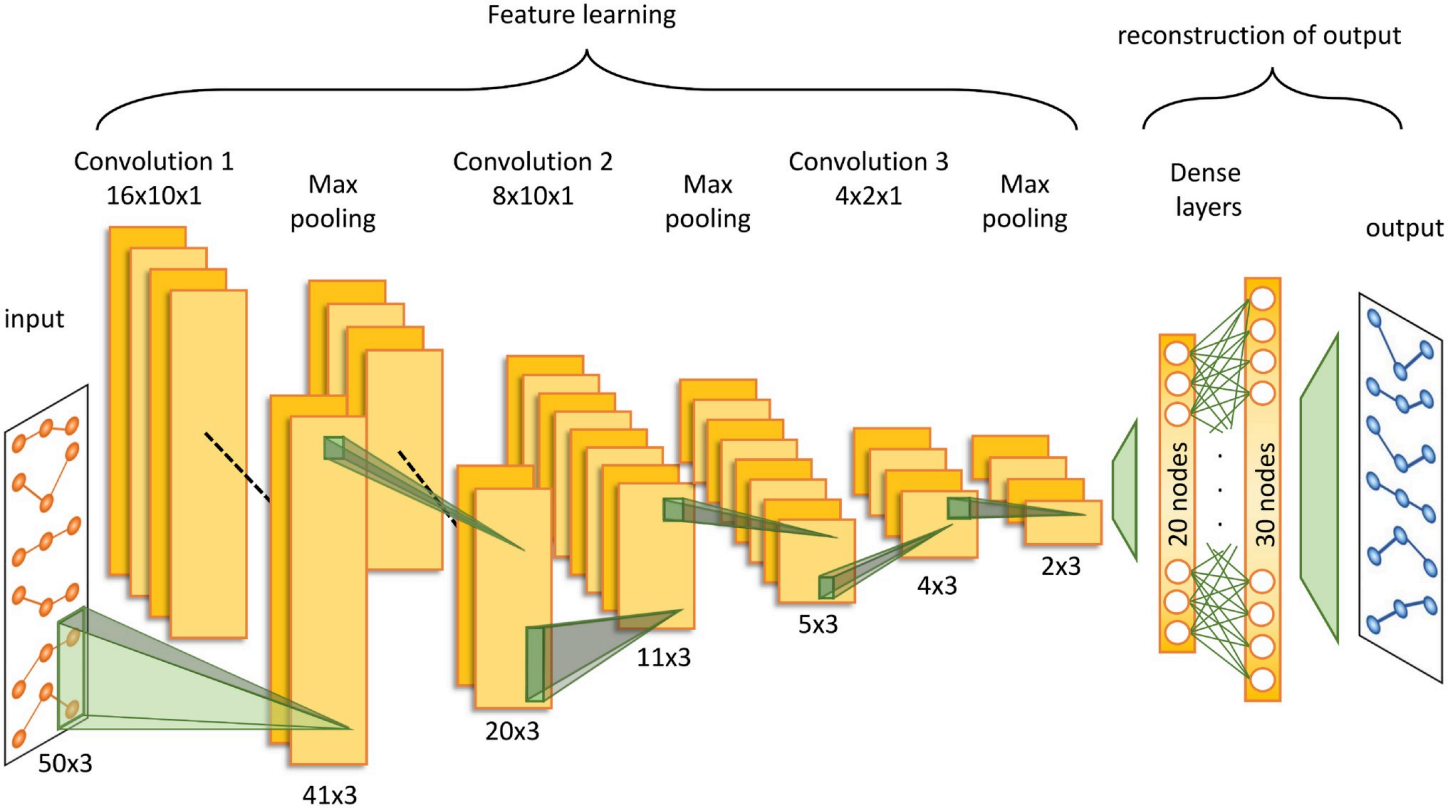
Outline of the structure of the implemented convolutional neural network. The convolutional neural network consists of three alternating convolutional and MaxPooling layers. The output from the final MaxPooling layer is then flattened into a vector which then passed through two fully connected layers to reconstruct the time series of gene expression pattern in the target domain. The above figure illustrates the rat *in vitro* to human *in vitro* prediction for the steatosis gene sets consisting of 50 genes. The gene expression data is reconstructed into a 2D format, with the genes on one axis and the respective time points on the other yielding an input of 50 genes by 3 time points as depicted in the diagram.

The architecture of convolutional neural networks (CNNs) is analogous to the connectivity pattern in the visual cortex, with neurons responding to stimuli in only a limited region of the visual field [41]. As the filters applied in the convolutional layers look at local regions, CNNs greatly reduce the number of weights requiring training. For input to the CNN model, the gene expression data was reshaped into a 2D format, with genes on one axis and corresponding time points on the other. CNNs consist of alternating convolutional and pooling layers followed by fully connected layers [42]. In our network, Fig 5, the first convolutional layer consists of 16 filters with a 10x1 kernel, followed by MaxPooling [43] layer of pool size 2x1. This is followed by an 8-filter convolution again with a 10x1 kernel, with a second MaxPooling layer. A third convolutional layer uses four filters with a 2x1 kernel, followed by a third MaxPooling layer. The output of the final MaxPooling layer is then flattened to a vector which passed into a 20 node, fully connected layer with L1 activity regularisation. The output is then up-sampled using a 30 node fully connected layer, followed by a fully connected output layer.

All deep learning models are trained using Adam [44], an adaptive stochastic gradient descent algorithm.

#### Machine learning models

In the absence of a gold standard in the field, in order to evaluate the added benefit of using deep learning models to predict time series of gene expression, classical machine learning techniques were used as a benchmark. Random Regression Forest (RRF) [45] and k-nearest neighbours (KNN) [46] were selected as our bench-marking models. This bench-marking strategy is in line with the validation methods used in previous studies to assess the added value of deep learning approaches on genomics data [25, 28].

## Results

Three deep learning models (Naïve Encoder, Modified Autoencoder and CNN) are evaluated for predicting time series of gene expression in a target domain (human *in vitro* or rat *in vivo*) given a measured time series of rat *in vitro* gene expression following exposure to a previously unseen compound.

### Across-species prediction—Rat *in vitro* to human *in vitro*

The average mean absolute error for the model predictions of time series of human *in vitro* gene expression given rat *in vitro* gene expression for the models included in this analysis are summarised in Table 1. The Naïve Encoder and CNN consistently outperformed both bench-marking methods (KNN and RRF) for all four toxicologically relevant gene sets included in this study. In fact, the CNN model has a significantly reduced average mean absolute error when compared to the RRF for each gene set, calculated using a paired two-tailed t-test with Benjamini-Hochberg correction for multiple testing within each gene set. The KNN model was consistently the poorest performing model across all gene sets. It is also noteworthy that the average mean absolute error for predicting time series of human *in vitro* gene expression decreases as the number of included genes increases for all methods. The average mean absolute error for the CNN model decreases from 0.0238 units for the cholestasis gene set (18 genes) to 0.01658 units for the GTX+C gene sets(76 genes). Suggesting the additional information added by including more genes improves the model predictions.

**Table 1:** Average mean absolute error from leave one out cross validation for each model predicting the four toxicologically relevant gene sets identified from literature.

Gene set	ref.	Cholestasis	NAFLD	Steatosis	GTX + C
number of genes		18	22	50	76
k-nearest-neighbour	[46]	0.02836	0.02303	0.02270	0.02076
random regression forest	[45]	0.02335	0.0183	0.01865	0.01718
naive encoder	[37]	0.02326	0.01819	0.01862	0.01667*
modified autoencoder	[40]	0.02507	0.01987	0.01972	0.01768
CNN	[41]	0.02138*	0.01742*	0.01841*	0.01658*

Table 1 shows the validation errors of the five models included in this analysis (rows) for predicting human *in vitro* gene expression given rat *in vitro* gene expression for each of the four toxicologically relevant orthologous gene sets identified from literature. The validation errors are the average of the mean absolute errors (mean prediction error across genes) calculated during the leave one out cross validation for the 45 compounds included in this study. The number of genes included in each gene sets is indicated in the second row. Emboldened values indicate the best performing model for each gene set.

* indicated models that have a significantly lower average mean absolute error than random regression forest for that gene set as calculated using a paired two-tailed t-test with correction for multiple testing using Benjamini-Hochberg correction.

**Fig 6:**
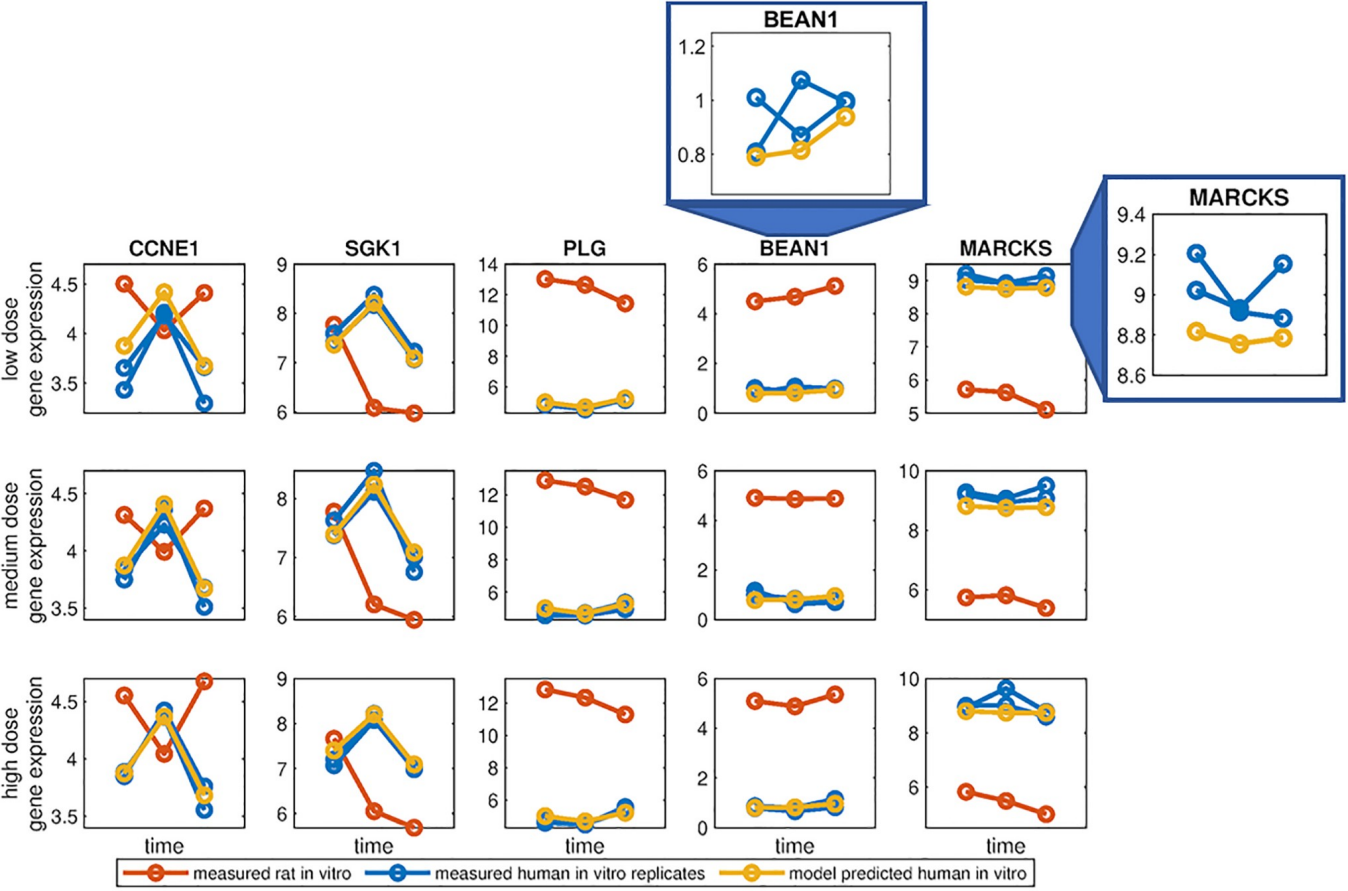
Input data and model predictions for validation compound hexachlorobenzene for convolutional neural network trained on carcinogenicity/genotoxicity gene set. The measured time series of rat *in vitro* (red) and human *in vitro* (blue) gene expression for a subset of genes from the GTX+C gene set for a low, medium, and high dosage of hexachlorobenzene are visualised along each row. A visual representation of the model predictions of a time series of human *in vitro* gene expression (yellow) relative to measured human *in vitro* gene expression in both biological replicates (blue) for the same subset of genes from the GTX/C gene set. The model prediction and human *in vitro* biological replicates for BEAN1 and MARCKS are visualised on a closer scale for the low dose exposure.

Fig 6 visualises the prediction of human *in vitro* gene expression for a subsets of genes from the GTX/C gene set using the CNN model for the low, medium, and high dosages of the validation compound hexachlorobenzene, a visusal representation for the cholestasis, NAFLD, and Steatosis gene sets are provided in the supplementary material (Fig S3a, S3b, S3c in S1 File). In each row the measured time series of gene expression for *in vitro* rat (red) and both human *in vitro* replicates (blue) following an exposure are visualised. The rat *in vitro* and human *in vitro* gene expression often notably differ in pattern (CCNE1 and SGK11) demonstrating the potential issues of relying on orthologs to directly translate gene expression patterns between species. The model predicted time series of human *in vitro* gene expression are depicted in yellow. In instances where the measured human *in vitro* replicates have inconsistent gene expression patterns the model predicts the mean gene expression pattern, as evident when zooming in on the model predictions for BEAN1 and MARCKS for a low dose of hexacholorbenzene in Fig 6. A visualisation of the CNN model prediction for the full GTX/C gene set following a low, medium, and high dose exposure to hexachlorobenzene is provided in Supplementary Figs S4a, S4b, and S4c in S1 File.

### Across experimental technique—Rat *in vitro* to rat *in vivo*

The above analysis was repeated to evaluate the potential for the deep learning models to also predict time series of *in vivo* gene expression given *in vitro* gene expression following an exposure in rats. The average mean absolute errors for the model predictions of time series of rat *in vivo* gene expression are summarised in Table 2. Again, the CNN and the Naïve Encoder consistently outperform the classical machine learning methods used for benchmarking, although the RRF does have a lower average mean absolute error than the Naïve Encoder for the cholestasis gene set. While the average mean absolute error values are greater than the human *in vitro* predictions, the error values again decrease as the size of the gene set increases for all models.

**Table 2:** Average mean absolute error from leave one out cross validation for each model predicting rat *in vivo* gene expression from rat *in vitro* for the four toxicologically relevant gene sets.

Gene set	ref	Cholestasis	NAFLD	Steatosis	GTX + C
number of genes		18	22	50	76
k-nearest-neighbour	[46]	0.04991	0.04027	0.03643	0.03140
random regression forest	[45]	0.03934	0.03188	0.02883	0.02479
naive encoder	[37]	0.03977	0.03102	0.02859	**0.02437**
modified autoencoder	[40]	0.04233	0.03438	0.03056	0.02626
CNN	[41]	0.03771*	0.03040*	**0.02849**	0.02462

Table 2 shows the validation errors of the models included in this analysis (rows) for predicting rat *in vivo* gene expression given rat *in vitro* gene expression for each of the four toxicologically relevant orthologous gene sets identified from literature. The validation errors are the average of the mean absolute errors (mean prediction error across genes) calculated during the leave one out cross validation for the 45 compounds included in this study. The number of genes included in each gene sets is indicated in the second row. Emboldened values indicate the best performing model for each gene set.

* indicated models that have a significantly lower average mean absolute error than random regression forest for a given gene set as calculated using a paired two-tailed t-test with correction for multiple testing using Benjamini-Hochberg correction.

**Fig 7:**
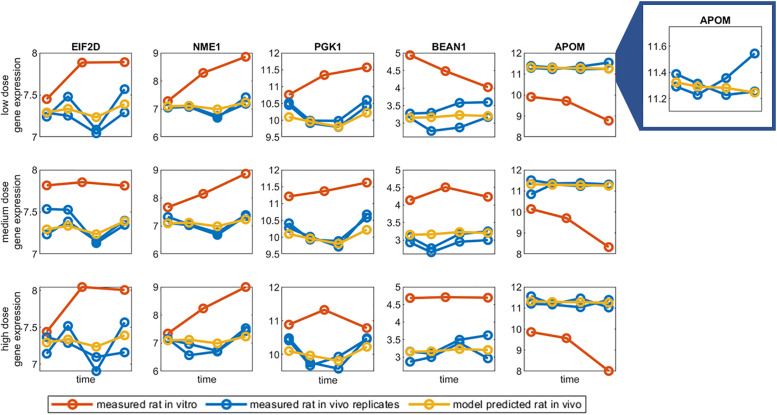
Input data and model predictions for validation compound azathioprine for convolutional neural network trained on carcinogenicity/genotoxicity gene set. The measured time series of rat *in vitro* (red) and rat *in vivo* (blue) gene expression and the model predictions of rat *in vivo* gene expression (yellow) for a subset of genes from the GTX/C gene set for a low, medium, and high dose exposure of azathioprine. The model prediction and both measured rat *in vivo* biological replicates for APOM are visualised on a closer scale for the low dose exposure to demonstrate the discrepancy in gene expression patterns that often exists for the rat *in vivo* biological replicates.

Fig 7 visualises the prediction of rat *in vivo* gene expression for a subset of genes from the GTX/C gene set using the CNN model for a low, medium, and high dose exposure the previously unseen compound azathioprine. As with the rat and human *in vitro* data, the measured time series of rat *in vitro* (red) and rat *in vivo* (blue) gene expression often differ in pattern (Fig 7). The model predicted time series of rat *in vivo* gene expression in visualised in yellow. There is a greater variance in gene expression patterns between the biological replicates for rat *in vivo* than observed in the human *in vitro* (Fig 7). As in the cross species predictions, the model often predicts the mean gene expression when the rat *in vivo* biological replicates differ in gene expression patterns (Fig 7 BEAN1 and APOM). A graphical representation the CNN model prediction for all 76 genes in GTX/C gene set following a low, medium, and high dose exposure to azathioprine is presented in Supplementary Figs S5a, S5b, and S5c in S1 File respectively. Visualisations of the model prediction for rat in vivo gene expression following a low dose of azathioprine for the Cholestatisis, NAFLD, and Steatosis gene sets can also be found in the supplementary material (Figs S6a, S6b, S6c in S1 File).

### Effect of gene set composition on model predictions

**Fig 8:**
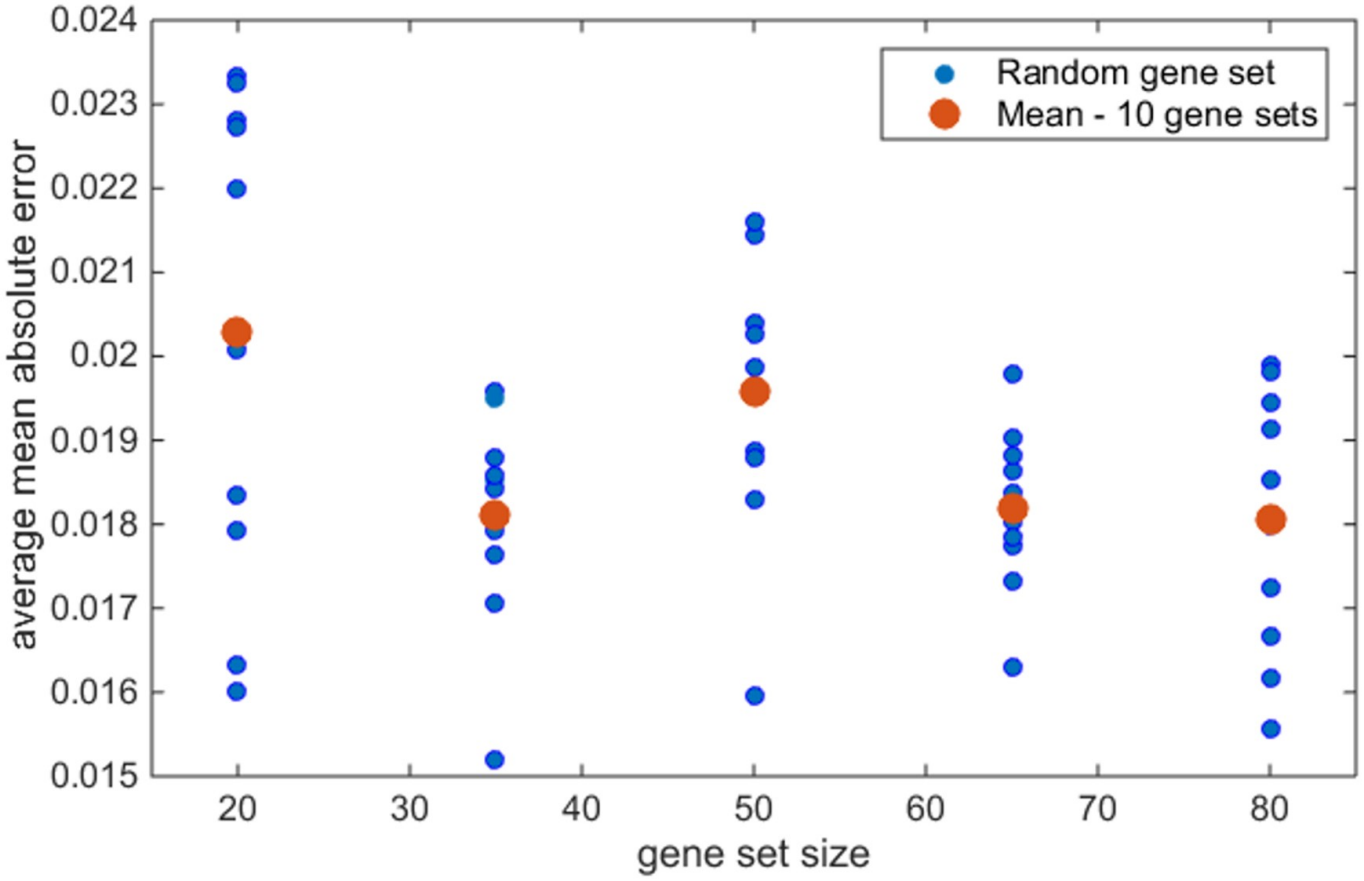
Average mean absolute error for randomly generated gene sets of increasing size for convolution neural network. Average mean absolute error (AMAE) for CNN trained on ten randomly generated non-orthologous gene sets of increasing size (20, 35, 50, 65, and 80 genes) (blue). The average AMAE for each size of gene set is depicted in orange. Note the distribution of AMAE values for the ten gene sets of each size.

The toxicologically relevant gene sets identified from literature differed not only in gene composition but also in terms of the numbers of included genes. To evaluate the impact of gene set composition on the accuracy of the cross species prediction of human *in vitro* gene expression from rat *in vitro* gene expression each model was trained using leave-one-out cross validation for ten randomly selected gene sets of increasing size (20, 35, 50, 65, and 80 genes) reflecting the size of the toxicologically relevant gene sets identified from literature. The gene sets were screened to ensure minimal overlap between sets of equal size. There was a considerable variation in average mean absolute error across the ten randomly selected gene sets for all models (Fig 8). While the variation decreased, somewhat, as the number of genes increased, it was still marked. Training of each model ten separate times on the same gene set demonstrated that the large variation in prediction accuracy was not due to the randomisation in model initialisation (not shown). This establishes that the particular genes included in a gene set can have a profound effect on the resulting model prediction.

**Fig 9:**
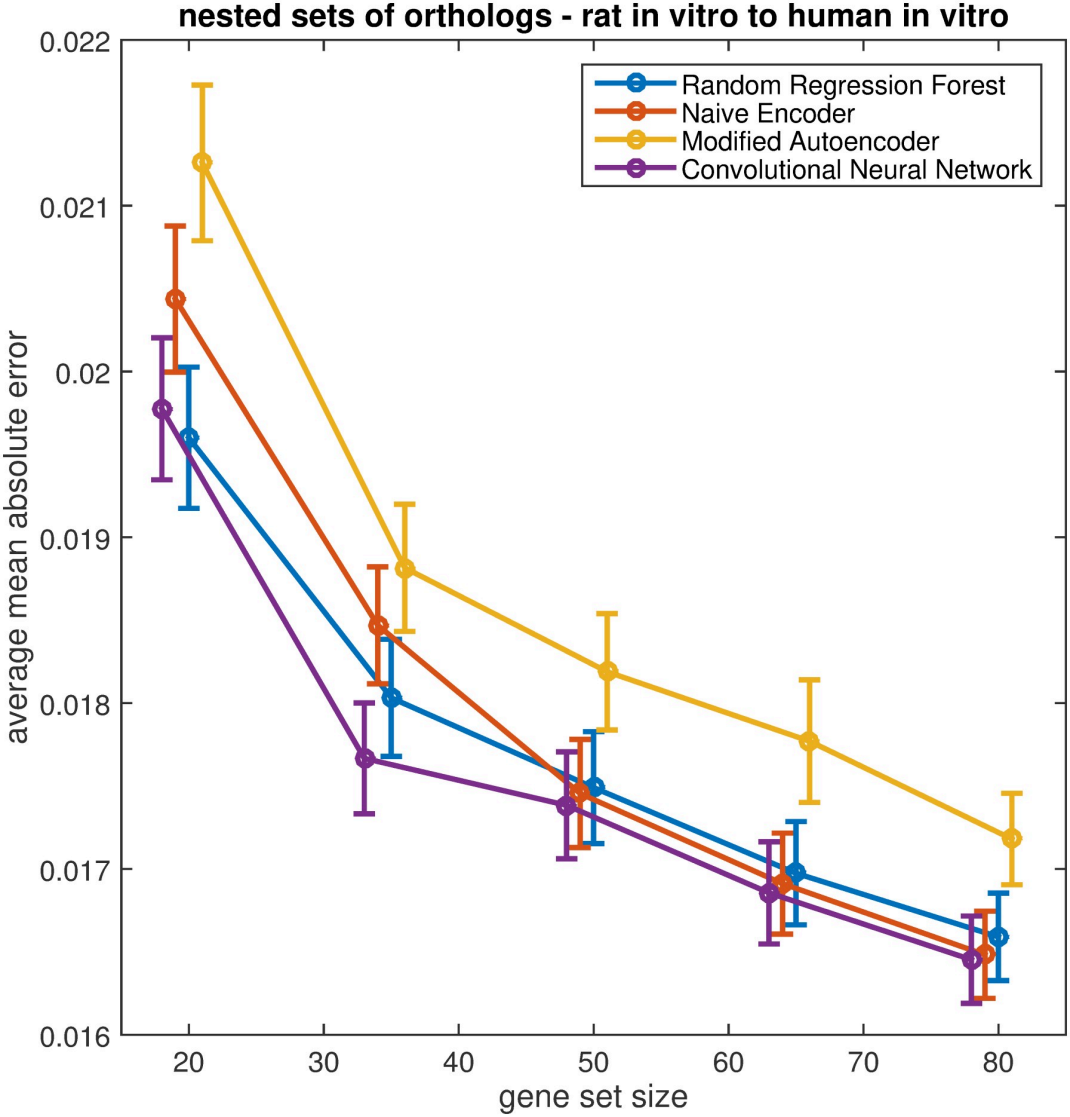
Average mean absolute error for each model trained on several nested sets of randomly selected genes of increasing size. Each model included in the analysis (CNN, naïve encoder, modified autoencoder, and random regression forest) were trained on a population of randomly selected nested gene sets of increasing size (20, 35, 50, 60, 80 genes). The figure depicts the mean average mean absolute error for each model trained on a population of thirty randomly generated non-orthologous gene sets of each size. The error bars indicate the standard error of the mean.

As each random gene set was generated independently, it was not possible to draw strong conclusions on the impact of the number of genes on the model predictions. To this end, 30 nested gene sets of increasing size were randomly generated, providing a more thorough analysis of the impact of added information (increasing number of genes) on the models’ predictions. The mean validation error for all models decreases as more genes are added (Fig 9). This reflects the results obtained for the toxicologically relevant gene sets, with GTX+C, the largest gene set, yielding the lowest average mean absolute error for all five models. *The above analysis was repeated for randomly selected orthologus gene sets from the rat in vitro to human in vitro prediction and also the in vivo predictions and gave comparable results (Supplementary material section 2). There appears to be no benefit is using only orthologus genes*.

## Discussion

The advent of genomics technologies, coupled with the growing pressure to reduce the need for animal testing, has contributed to a surge in the development of *in vitro* toxicogenomics bio-assays. While the identification of genomic signatures of adverse toxicological outcomes have yielded positive results in the classification of novel compounds [13–15], relating changes in genomic profile across species or from *in vitro* to *in vivo*, particularly for biological interpretation of underlying mechanisms still proves challenging not only in toxicological research, but also in several other biological disciplines that utilise cellular and animal models. In this study, we demonstrate the potential of deep learning methods to translate time series of drug-induced changes in gene expression across species and experimental domains. The deep learning models evaluated in this study outperform classical machine learning algorithms in predicting time series of human *in vitro* and rat *in vivo* gene expression given a measured time series of rat *in vitro* gene expression following an exposure to a previously unseen compound.

Of the three deep learning models included in this analysis the Naïve Encoder, a fully connected artificial neural network with a bottleneck architecture, and the CNN have the lowest average mean absolute error in both the cross-species and *in vitro* to *in vivo* predictions, consistently outperforming the Random Regression Forest, the best performing traditional machine learning method. CNN are increasingly being investigated for their potential in the analysis of gene expression data [47, 48]. Gene expression is a highly complex, co-regulated system, which is currently not fully understood. The kernels implemented in our CNN model have been restricted to filtering several genes at a single time point, identifying patterns of co-expression. By integrating further biological knowledge, it may be possible to apply a more logical structure to the genes in the input data, with neighbouring genes sharing some regulatory relationship. In this way, the local convolution may be more optimally utilised to filter for dependencies in neighbouring genes, improving the prediction accuracy.

Our Modified Autoencoder had consistently low prediction accuracies, out-performing only the KNN method. Use of the weights obtained by pre-training two independent autoencoders to initialise the prediction model may have hindered its training. A recent study applied domain adaptation to combine the pre-trained encoder and decoder weights [49], integration of this approach in the training of the network may improve the performance of our Modified Autoencoder.

As the number of genes included in the analysis increases, the accuracy of the predictions improves for all models. This is perhaps not surprising as the inclusion of additional genes increases the information from which the models can learn. While the performance of all models, including the classical machine learning approaches, improve with the addition of genes the reduction in average mean absolute error is more pronounced for the CNN and Naïve Encoder, suggesting the deep learning models have an advantage in prediction for larger gene sets. The improved performance is evident in both the toxicologically relevant gene sets identified from literature and the randomly selected gene sets. We have constrained our model predictions to relatively small gene sets consisting of approximately twenty to eighty genes. While, Open TG-GATEs was selected as a pilot data set as it is a comparatively large toxicogenomics data. The number of learning examples that could be generated is far less than the 20,000 genes measured by microarray analysis, necessitating the use of subsets of genes. This does not necessarily hinder our results; the use of subsets of genes, or genomic signatures, to classify potential adverse outcomes is commonplace in toxicological research. In addition, we also do not see any benefit in restricting the cross species prediction task to orthologus genes, with the deep-learning models preforming equally as well on sets of randomly selected genes (Fig 9) as randomly selected sets of orthologs (Supplementary Fig S7 in S1 File).

In order to evaluate the impact of the number of genes on model predictions nested gene sets containing between twenty and eighty genes were generated. This range of values was selected to reflect the numbers of genes in our toxicologically relevant gene sets while also bearing in mind the 720 learning examples available for training. Generally, a ratio of 10 learning examples to number of features is advised, guiding the choice of gene set sizes included in these analyses. Looking at the average mean absolute error values for models trained on these randomly generated nested gene sets it is evident that model performance improves with the addition of more genes. However, we would expect this improvement in validation error to plateau at a certain gene set size as the dimensionality of the feature space would become too large for the limited number of learning examples. The models would then be expected to over-fit the training data and the validation error would increase. This point has not been identified in our analyses.

As this was a provisional study evaluating the potential of deep learning algorithms for this cross-domain prediction task it was decided to restrict training to a single data set of gene expression measurements removing variation in the data due to differing experimental protocols. TG-GATEs was selected as it is a large toxicogenomics data set. Sprague-Dawley rats were used for both *in vitro* and *in vivo* experiments. The primary human hepatocytes used in the experiments included in these analyses were derived from a single human donor. Consequently, the subset of TG-GATEs used here provides a somewhat an idealised scenario for evaluating the performance of the deep learning models greatly reducing variation in data due to experimental technique. Despite this the average mean absolute error values for the *in vivo* prediction are greater than the human *in vitro* prediction (Tables 1 and 2), we believe this may be largely attributed to the greater variation in the gene expression pattern for the rat *in vivo* replicates when compared to the human *in vitro* replicates. It may be possible to increase the number of genes included in the analysis and improve the accuracy of the models predictions by combining time series of gene expression from other toxicological data bases, such as DrugMatrix [50], thereby increasing the number of learning examples. The variation in gene expression introduced by the differing experimental protocols, and more genetically diverse hepatocytes may further improve the generalisability of the models.

Within the context of this study, we were primarily concerned with the predictive ability of the models. We have not explored the connection weights or structure of the latent space of the trained models. Exploration of the latent spaces of neural networks applied in other classification tasks has provided insight into how the networks learn and make decisions [26]. Due to the bottleneck structure in the networks implemented within this study the models are forced to learn a compressed representation of the data. Accordingly, the time series of gene expression predicted in the target domain is formed by a nonlinear combination of the input gene expression patterns of multiple genes. Exploration of the most influential features in the final, fully trained models, may provide new biological insight into the relationship between genes. In addition, this reduced dimensional latent space may provide a novel method for the classification of potential adverse outcomes as it is anticipated that compounds that trigger similar responses in gene expression would cluster together in the compressed latent space [51, 52].

We have demonstrated the ability of deep learning models to translate time series of gene expression across domains; predicting across species, from rat to human *in vitro*, and from *in vitro* to *in vivo* for rats. Our aim in future work would be to train a model to predict human *in vivo* gene expression. While the simple deep learning architectures evaluated in this study show potential, they require vast quantities of data to train, which would be unavailable for the human *in vivo* domain. It may be possible to further adapt the *in vitro* to *in vivo* models trained on rat data to the human task by integrating some form of transfer learning as has been utilised by Herdon and Caragea to predict protein localisation and splice site locations in a given organism by leveraging large volumes of publicly available labelled data from well studied model organisms [53, 54].

### Conclusions

We have demonstrated the potential of deep learning architectures, most notably Convolution Neural Networks and deep fully connected networks, in translating drug-induced changes in gene expression across the species and experimental domains. These deep learning models outperform classical machine learning algorithms in predicting time series of human *in vitro* and rat *in vivo* gene expression given a measured time series of rat *in vitro* gene expression following exposure to a novel compound. More importantly, we highlight the emerging potential of deep learning models in both the analysis of gene expression data and classification of toxicity.

## Supporting information

S1 File (PDF)

S1 Scripts (TXT)
