# Supplementary material for: Use of deep learning methods to translate drug-induced gene expression changes from rat to human primary hepatocytes
Source: PLoS One. 2020 Aug 11;15(8):e0236392. doi: 10.1371/journal.pone.0236392 (PMC7418976; doi:10.1371/journal.pone.0236392)
Supplement: S1 File — (PDF) [file pone.0236392.s001.pdf]

Use of deep learning methods to translate drug-induced gene expression changes from rat to human primary hepatocyte.

Shauna O'Donovan, Kurt Driessens, Daniel Lopatta, Florian Wimmenauer, Alexander Lukas, Jelmer Neeven, Tobias Stumm, Evgueni Smirnov, Michael Lenz, Gokhan Ertaylan, Danyel Jennen, Natal van Riel, Rachel Cavill, Ralf Peeters, Theo de Kok.

Supplementary material

Supplementary material Section 1 : Additional figures.

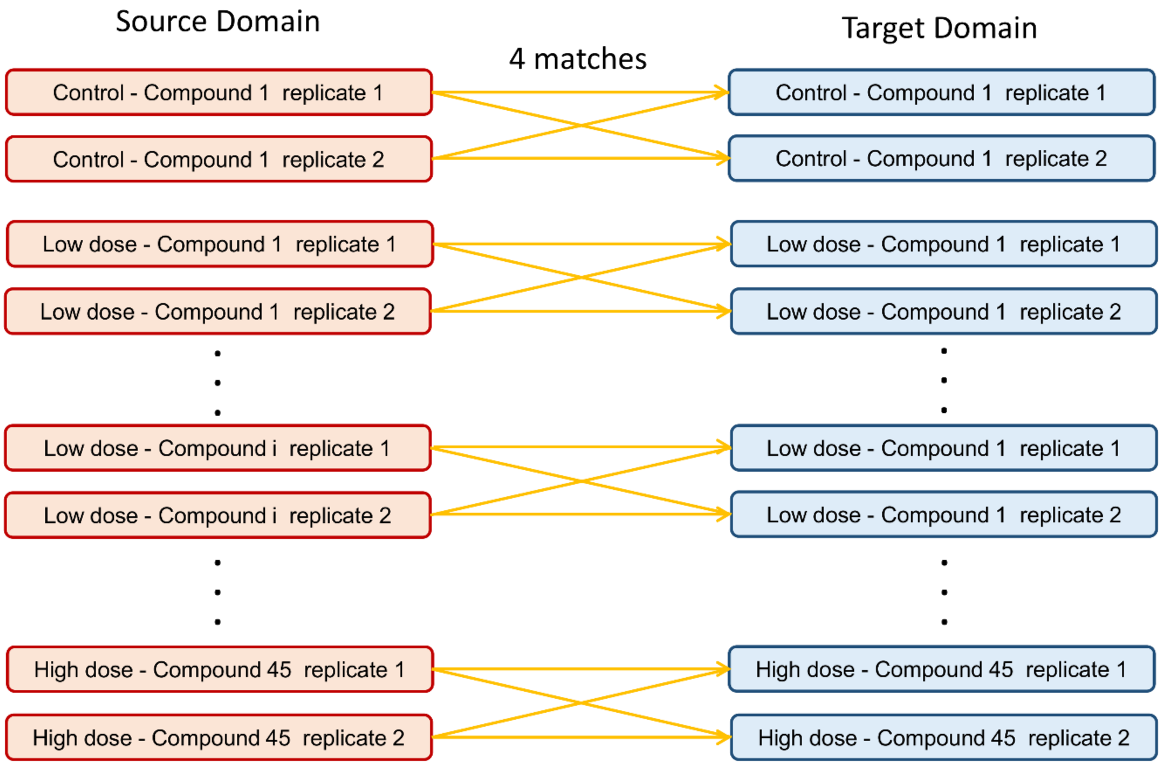

Figure S1 : Pairing strategy for generating machine learning examples.

Machine learning examples are generated by pairing the time series of gene expression in the source and target domain for each compound-dose combination. Both biological replicates for a given compound-dose combination in the target domain are a valid pair for each biological replicate for that compound-dose combination in the source domain. Pairwise matching of the biological replicates generates four machine learning examples for each compound-dose combination (illustrated with the yellow arrows above). Treating the controls as an additional dose; with 45 compound * 3 dosages plus the control * pairwise matching of biological replicates creates 720 learning examples.

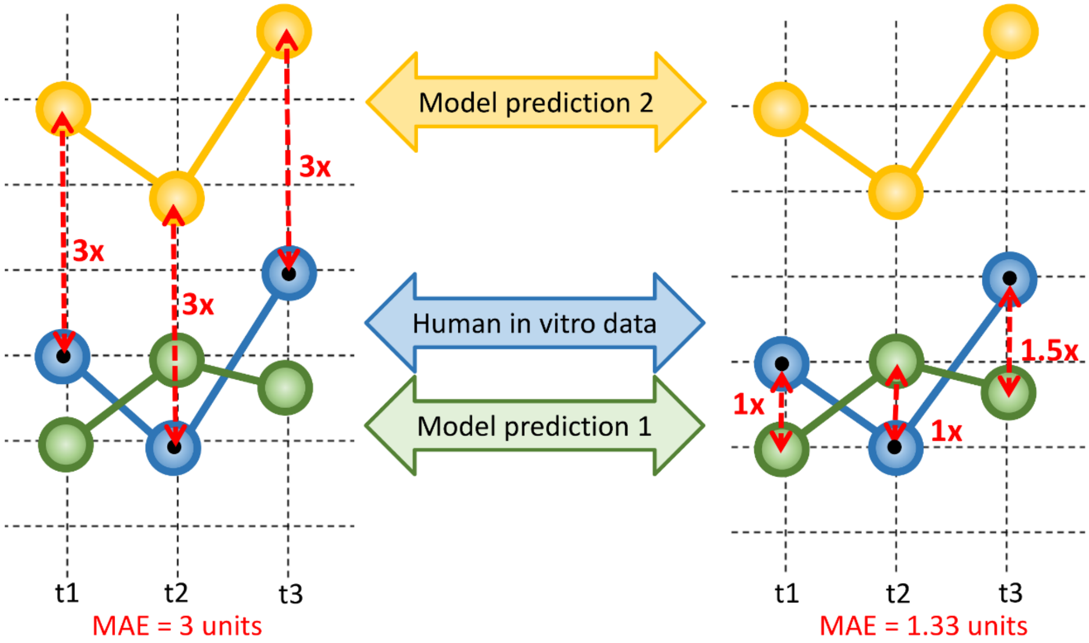

Figure S2a: Error calculation using mean absolute error for raw normalised gene expression values.

The above figure depicts two model predictions for a measured time series of human *in vitro* gene expression (blue). The gene expression values for model prediction 1 (green) are very close in magnitude to the measured time series of human *in vitro* gene expression, however, the gene expression pattern of Model prediction 1 differs greatly to the measure human *in vitro* gene expression pattern. Model prediction 2 (yellow) has the same gene expression pattern as the time series measured human *in vitro* gene expression, but it is shifted upwards. Evaluating the quality of the model predictions using a classical distance based cost function, such as sum of absolute errors, on the raw gene expression level values we see that model prediction 1 would have a much lower error value despite having such a different pattern in gene expression. Despite model prediction 2 predicting the correct gene expression pattern the penalty for incorrectly predicting the gene expression level at the first time point is also added to each subsequent time point.

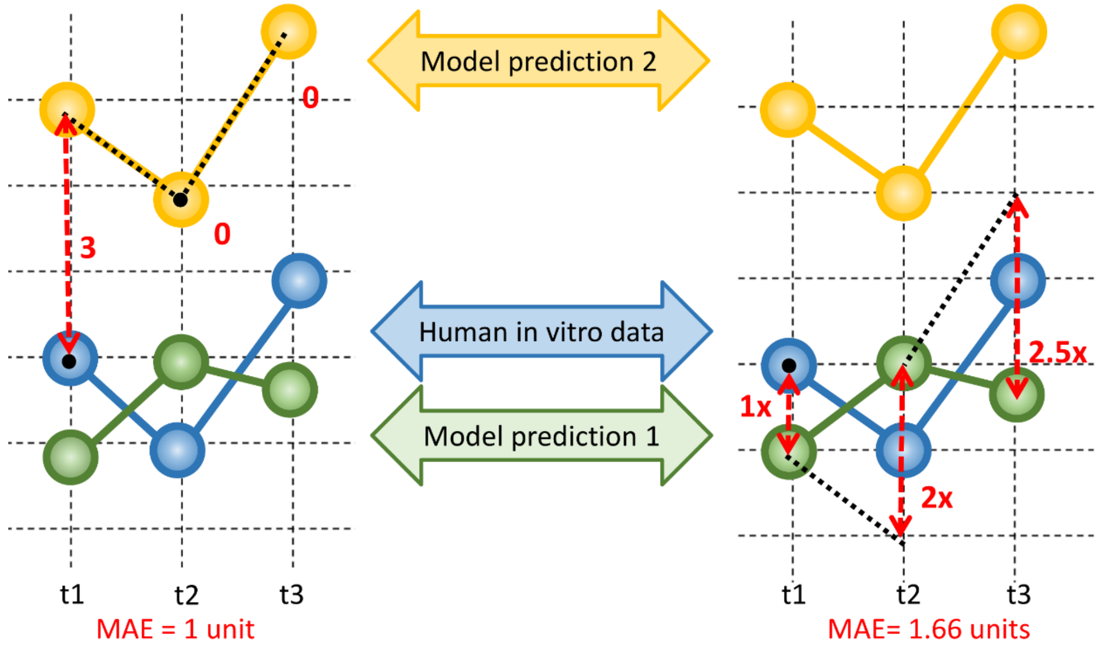

Figure S2b: Error calculation using mean absolute error for slope re-encoded gene expression values.

The gene expression data is re-encoded, the first entry remains unchanged, subsequent entries now encode the slope, change in gene expression levels, between consecutive time points. Applying the same error calculation as before (mean absolute error) we see that while model prediction 2 is penalized for incorrectly predicting the gene expression value for time point one as it correctly predicts the changes in gene expression between time point one and two, and time point two and three, it receives no further penalty. While model prediction 1 is closer in predicting the level of gene expression for time point one, and there receives a lower penalty, as if fails to predict the correct changes in gene expression level for subsequent time point it has a greater error over all. In this way, model predictions are not unduly penalized for incorrectly predicting a single time point. By re-encoding the data in this manner the models are training to correctly predict the gene expression pattern without the need to introduce a more complex error function.

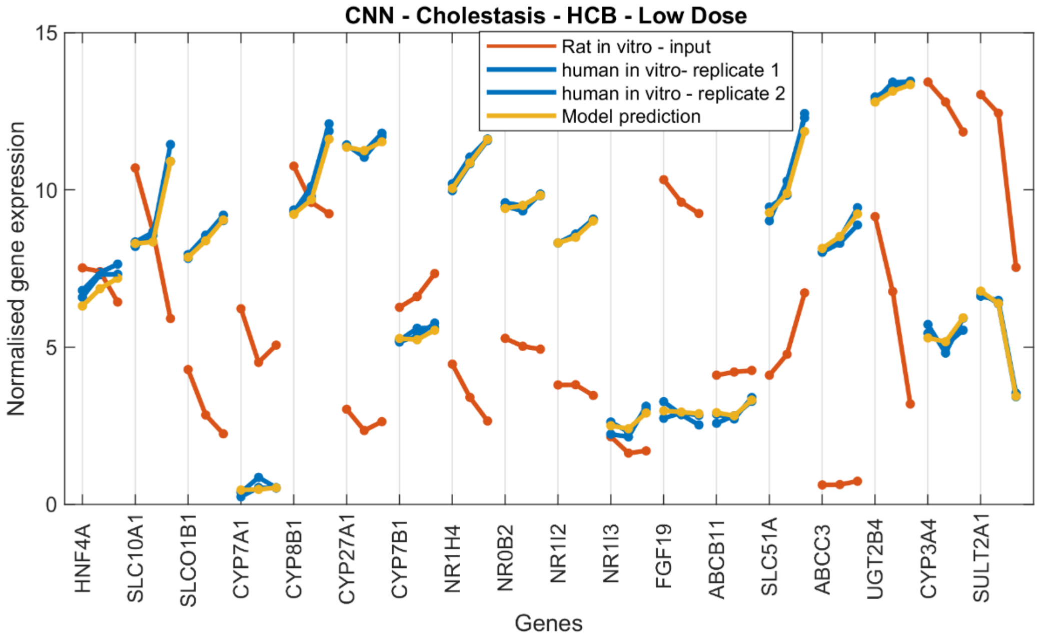

Figure S3a: Measured and model predicted time series of rat *in vitro* to human *in vitro* gene expression for the full Cholestasis gene set for the validation compound hexachlorbenzene (low dose) for the CNN model

Measured time series of rat *in vitro* gene expression (red) following an exposure to a low dose of the validation compound hexachlorobenzene is input to the CNN model. The model predictions of human *in vitro* gene expression are in yellow. Both biological replicates for the time series of human *in vitro* gene expression following low dose exposure of hexachlorobenzene are depicted in blue for reference. Data is shown for the cholestasis gene set (18 genes, known rat-human orthologs)

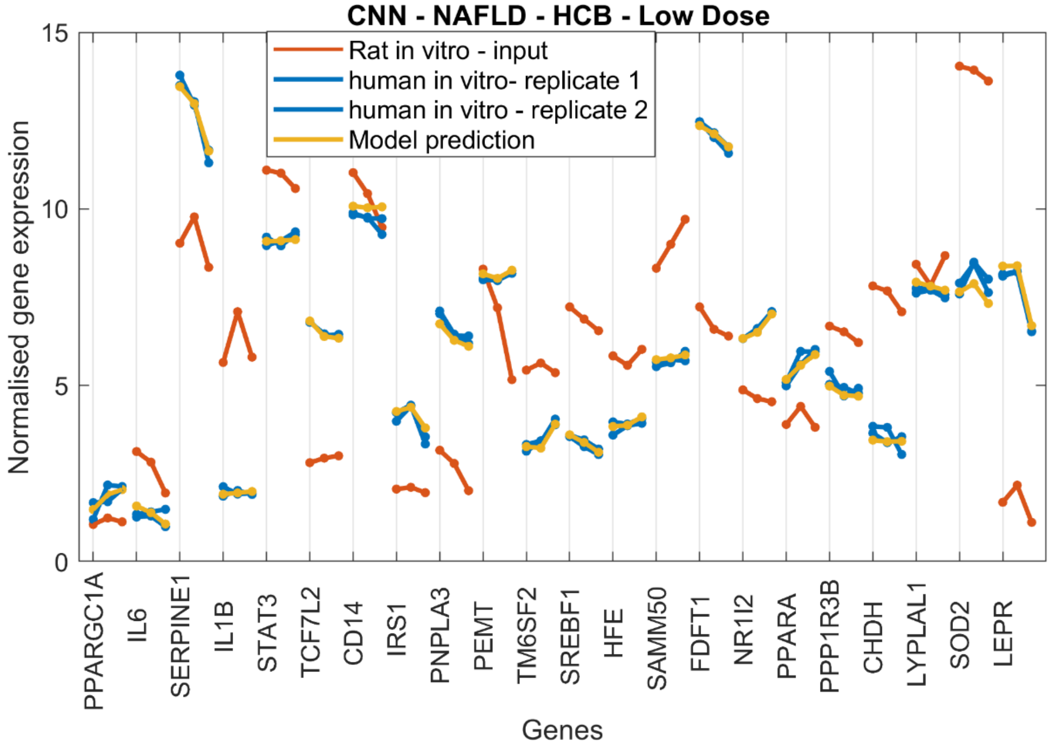

Figure S4b: Measured and model predicted time series of rat *in vitro* to human *in vitro* gene expression for the full NAFLD gene set for the validation compound hexachlorbenzene (low dose) for the CNN model

Measured time series of rat *in vitro* gene expression (red) following an exposure to a low dose of the validation compound hexachlorobenzene is input to the CNN model. The model predictions of human *in vitro* gene expression are in yellow. Both biological replicates for the time series of human *in vitro* gene expression following low dose exposure of hexachlorobenzene are depicted in blue for reference. Data is shown for the NAFLD gene set (22 genes, known rat-human orthologs)

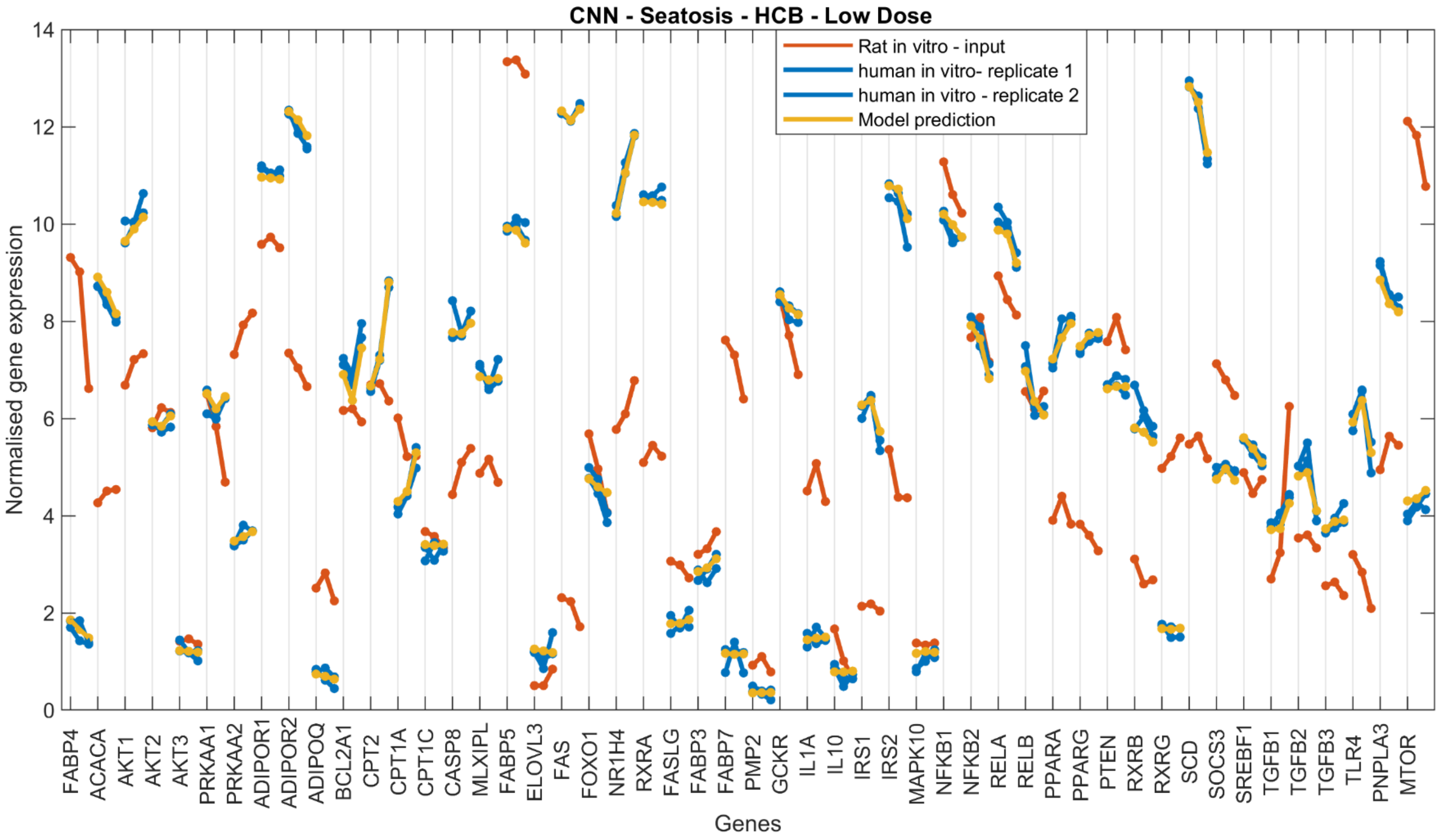

Figure S5c: Measured and model predicted time series of rat *in vitro* to human *in vitro* gene expression for the steatosis gene set for the validation compound hexachlorbenzene (low dose) for the CNN model

Measured time series of rat *in vitro* gene expression (red) following an exposure to a low dose of the validation compound hexachlorobenzene is input to the CNN model. The model predictions of human *in vitro* gene expression are in yellow. Both biological replicates for the time series of human *in vitro* gene expression following low dose exposure of hexachlorobenzene are depicted in blue for reference. Data is shown for the steatosis gene set (50 genes, known rat-human orthologs

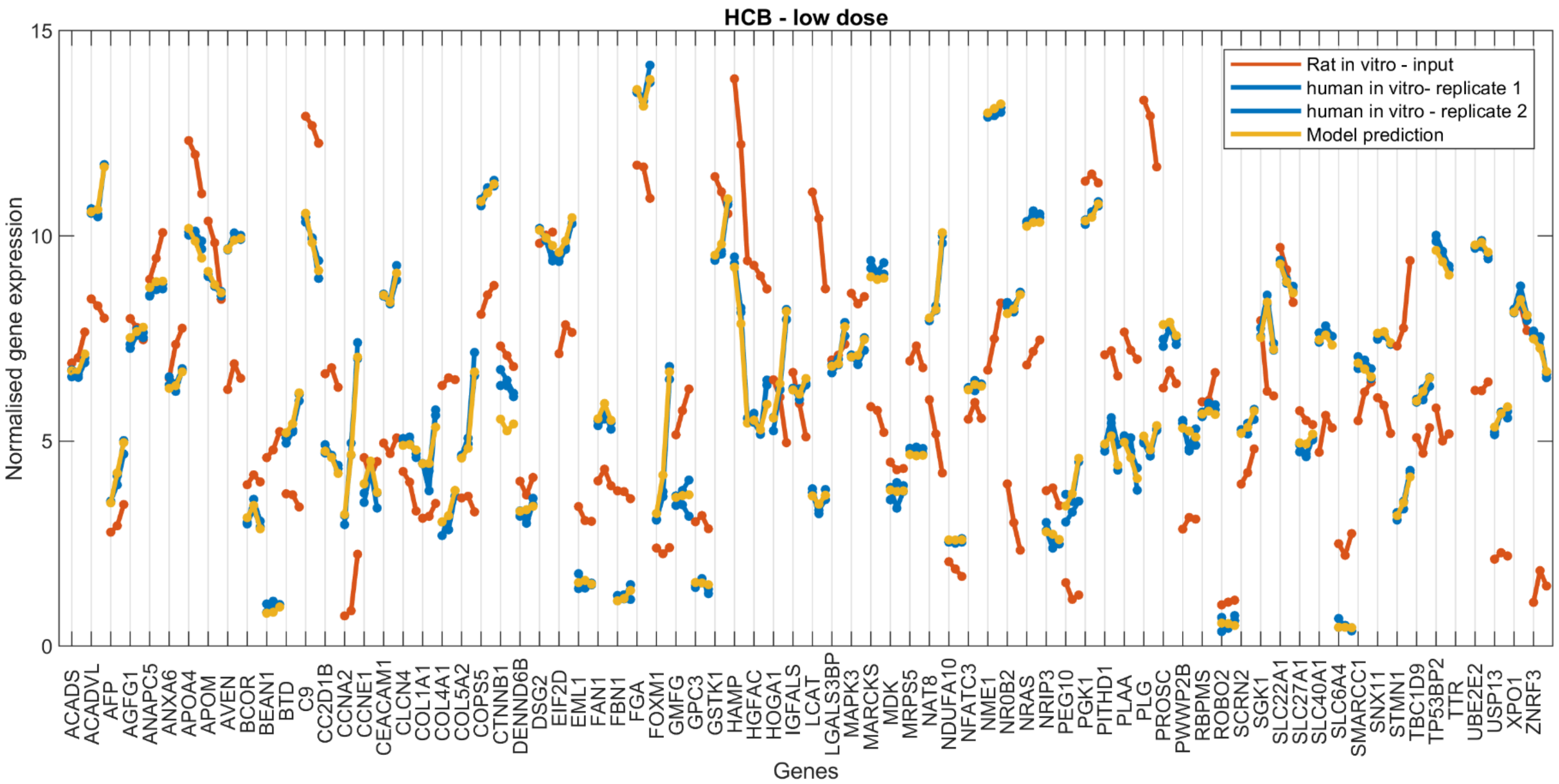

Figure S4a: Measured and model predicted time series of rat *in vitro* to human *in vitro* gene expression for the full GTX/C gene set for the validation compound hexachlorbenzene (low dose) for the CNN model

Measured time series of rat *in vitro* gene expression (red) following an exposure to a low dose of the validation compound hexachlorobenzene is input to the CNN model. The model predictions of human *in vitro* gene expression are in yellow. Both biological replicates for the time series of human *in vitro* gene expression following low dose exposure of hexachlorobenzene are depicted in blue for reference. Data is shown for the full GTX/C gene set (76 genes, known rat-human orthologs)

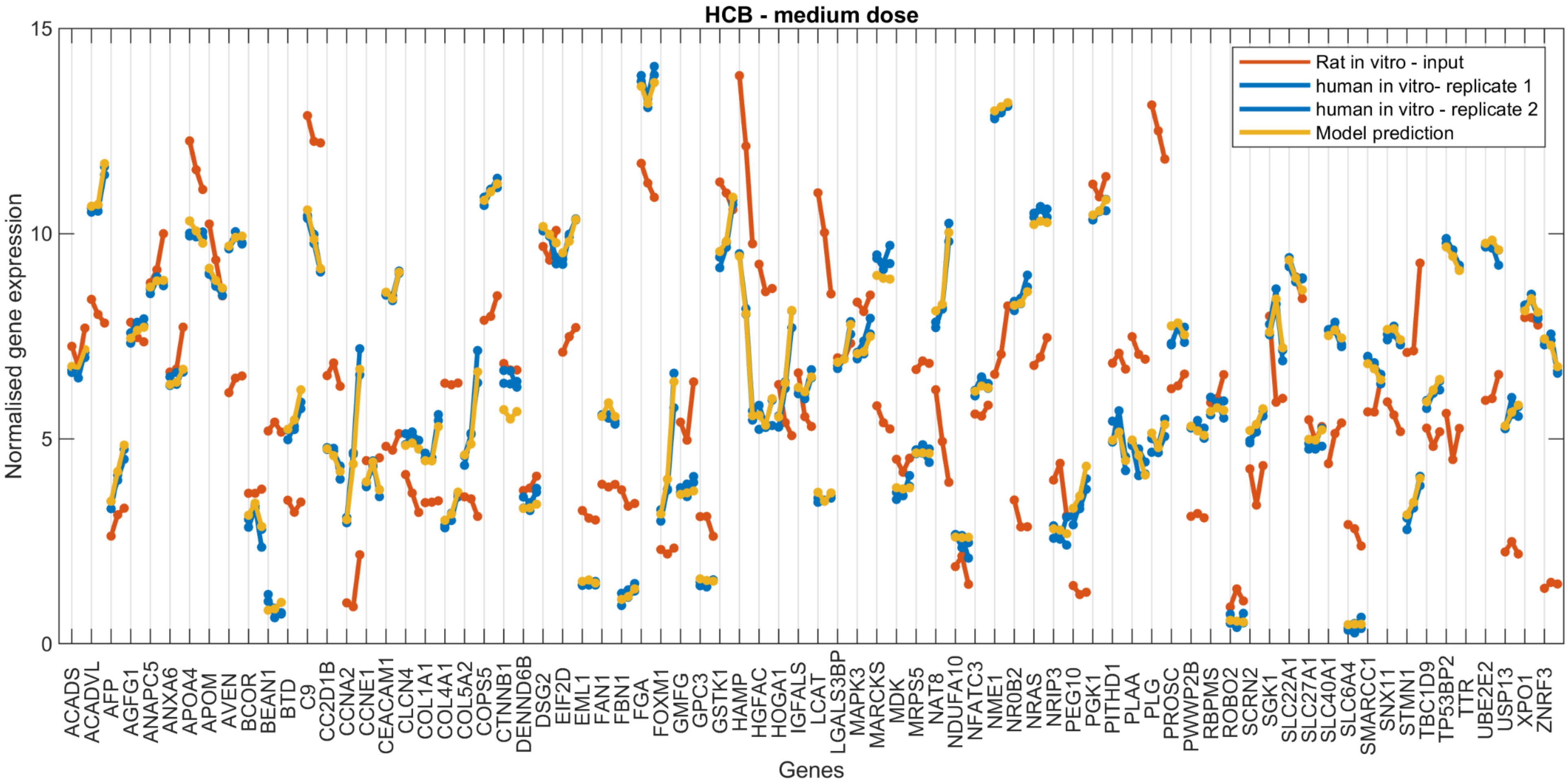

Figure S4b: Measured and model predicted time series of rat *in vitro* to human *in vitro* gene expression for the full GTX/C gene set for the validation compound hexachlorbenzene (medium dose) for the CNN model

Measured time series of rat *in vitro* gene expression (red) following an exposure to a medium dose of the validation compound hexachlorobenzene is input to the CNN model. The model predictions of human *in vitro* gene expression are in yellow. Both biological replicates for the time series of human *in vitro* gene expression following low dose exposure of hexachlorobenzene are depicted in blue for reference. Data is shown for the full GTX/C gene set (76 genes, known rat-human orthologs)

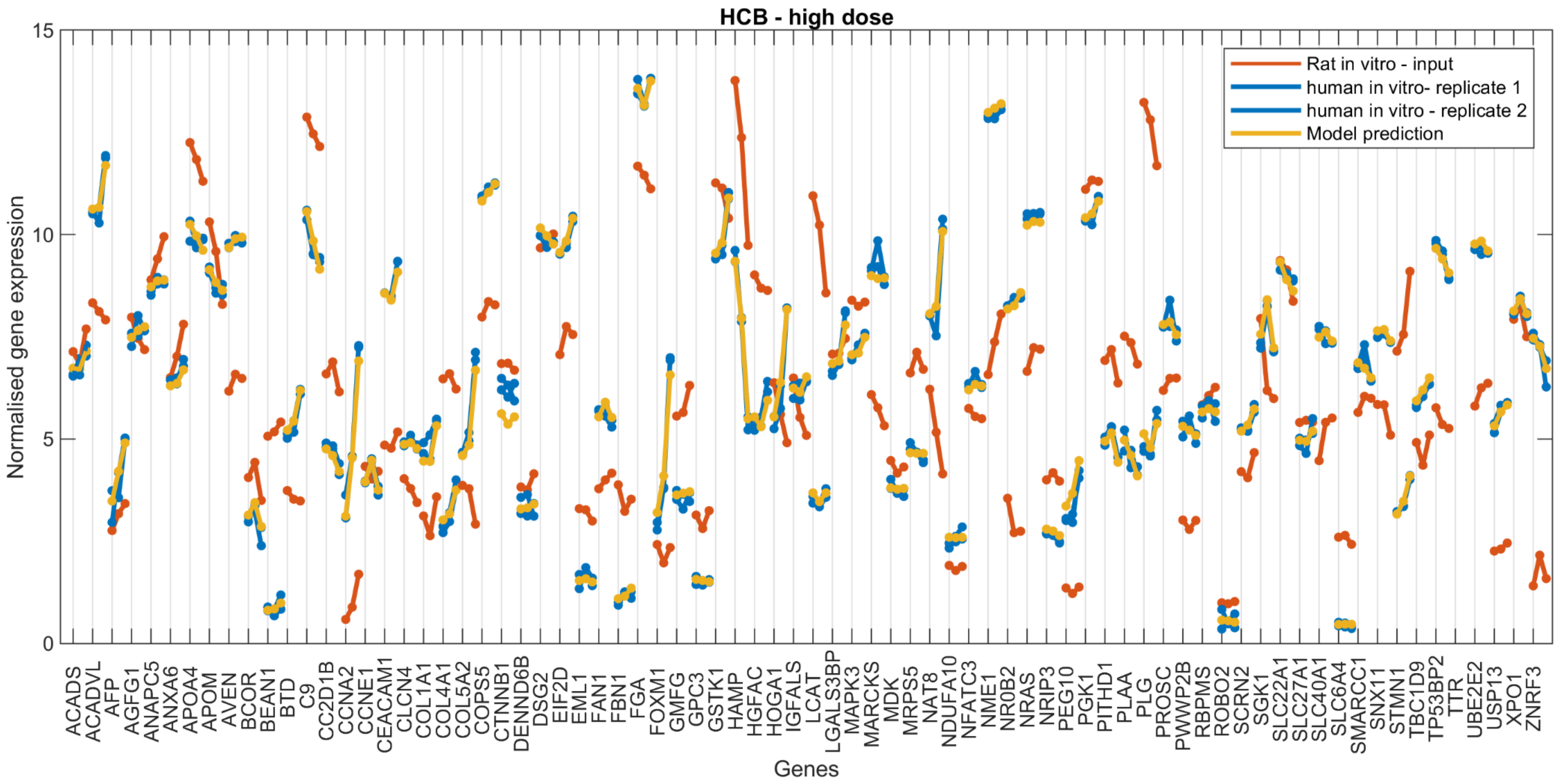

Figure S4c: Measured and model predicted time series of rat *in vitro* to human *in vitro* gene expression for the full GTX/C gene set for the validation compound hexachlorbenzene (highdose) for the CNN model

Measured time series of rat *in vitro* gene expression (red) following an exposure to a high dose of the validation compound hexachlorobenzene is input to the CNN model. The model predictions of human *in vitro* gene expression are in yellow. Both biological replicates for the time series of human *in vitro* gene expression following low dose exposure of hexachlorobenzene are depicted in blue for reference. Data is shown for the full GTX/C gene set (76 genes, known rat-human orthologs)

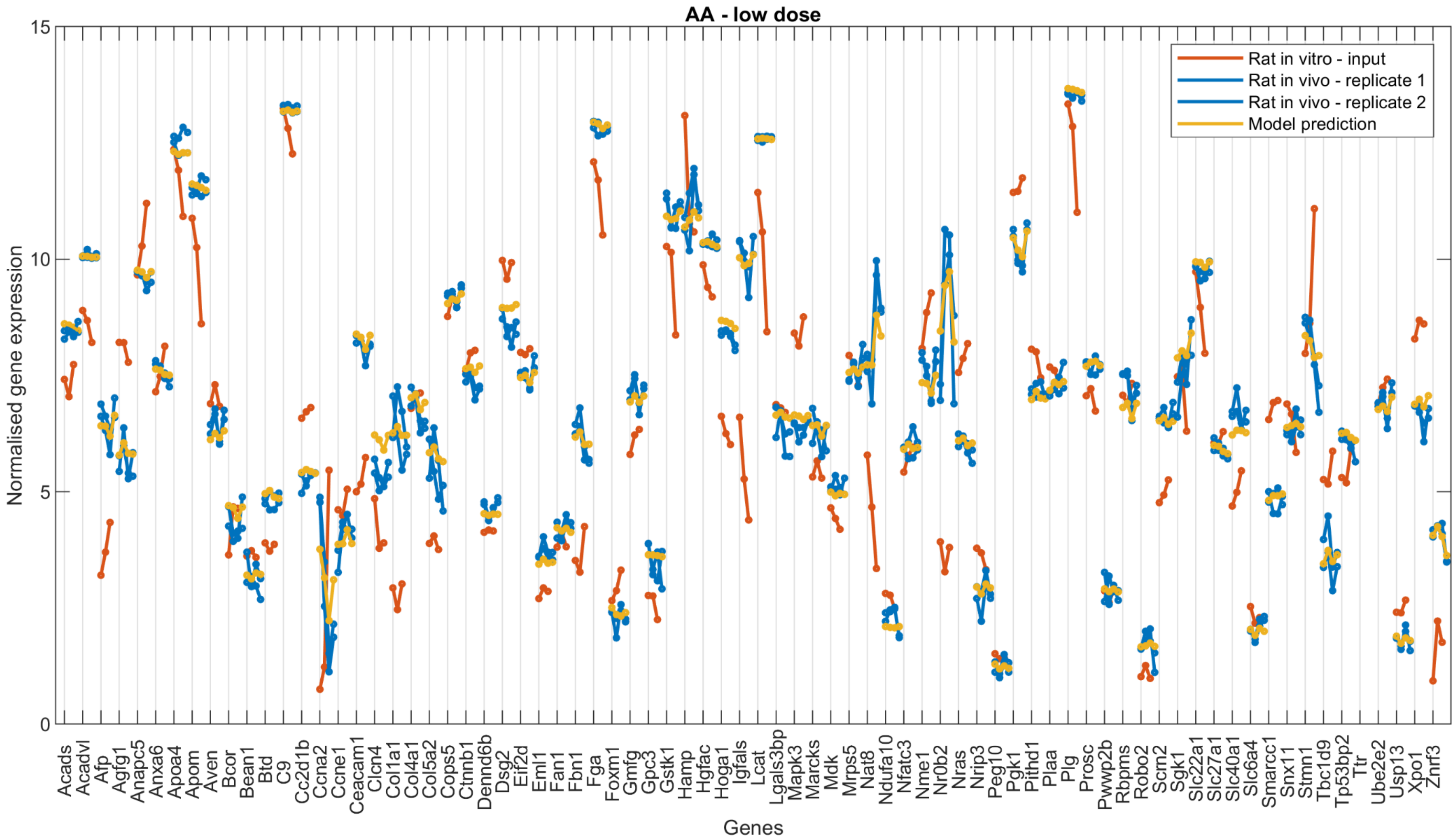

Figure S5a : Measured and model predicted time series of rat *in vitro* to rat *in vivo* gene expression for the full GTX/C gene set for the validation compound azathioprine (low dose) for the CNN model.

Measured time series of rat *in vitro* gene expression (red) following an exposure to a low dose of the validation compound azathioprine is input to the CNN model. The model predictions of rat *in vivo* gene expression are in yellow. Both biological replicates for the time series of rat *in vivo* gene expression following low dose exposure of azathioprine are depicted in blue for reference. Data is shown for the full GTX/C gene set (76 genes).

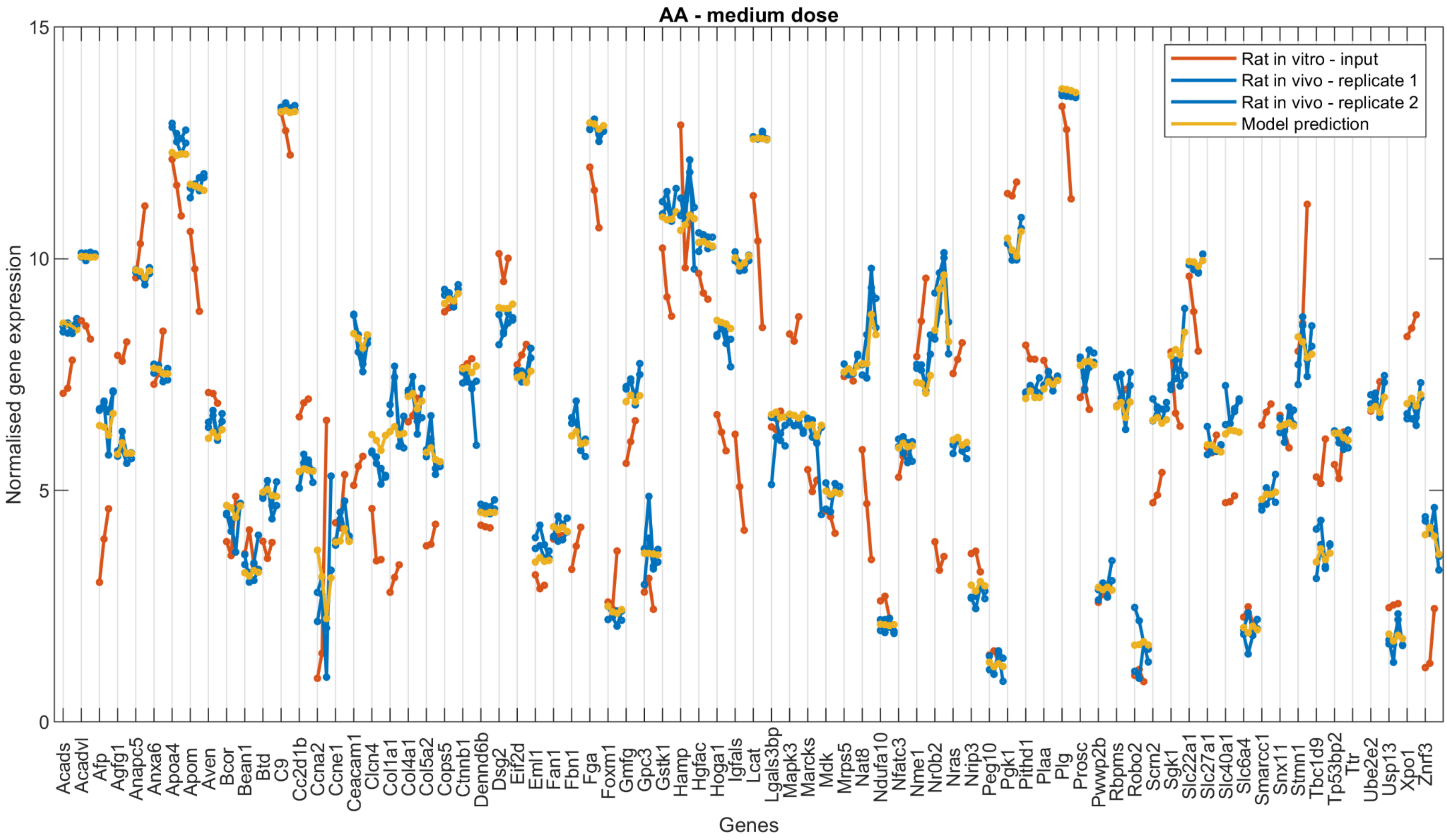

Figure S5b : Measured and model predicted time series of rat *in vitro* to rat *in vivo* gene expression for the full GTX/C gene set for the validation compound azathioprine (medium dose) for the CNN model.

Measured time series of rat *in vitro* gene expression (red) following an exposure to a medium dose of the validation compound azathioprine is input to the CNN model. The model predictions of rat *in vivo* gene expression are in yellow. Both biological replicates for the time series of rat *in vivo* gene expression following low dose exposure of azathioprine are depicted in blue for reference. Data is shown for the full GTX/C gene set (76 genes).

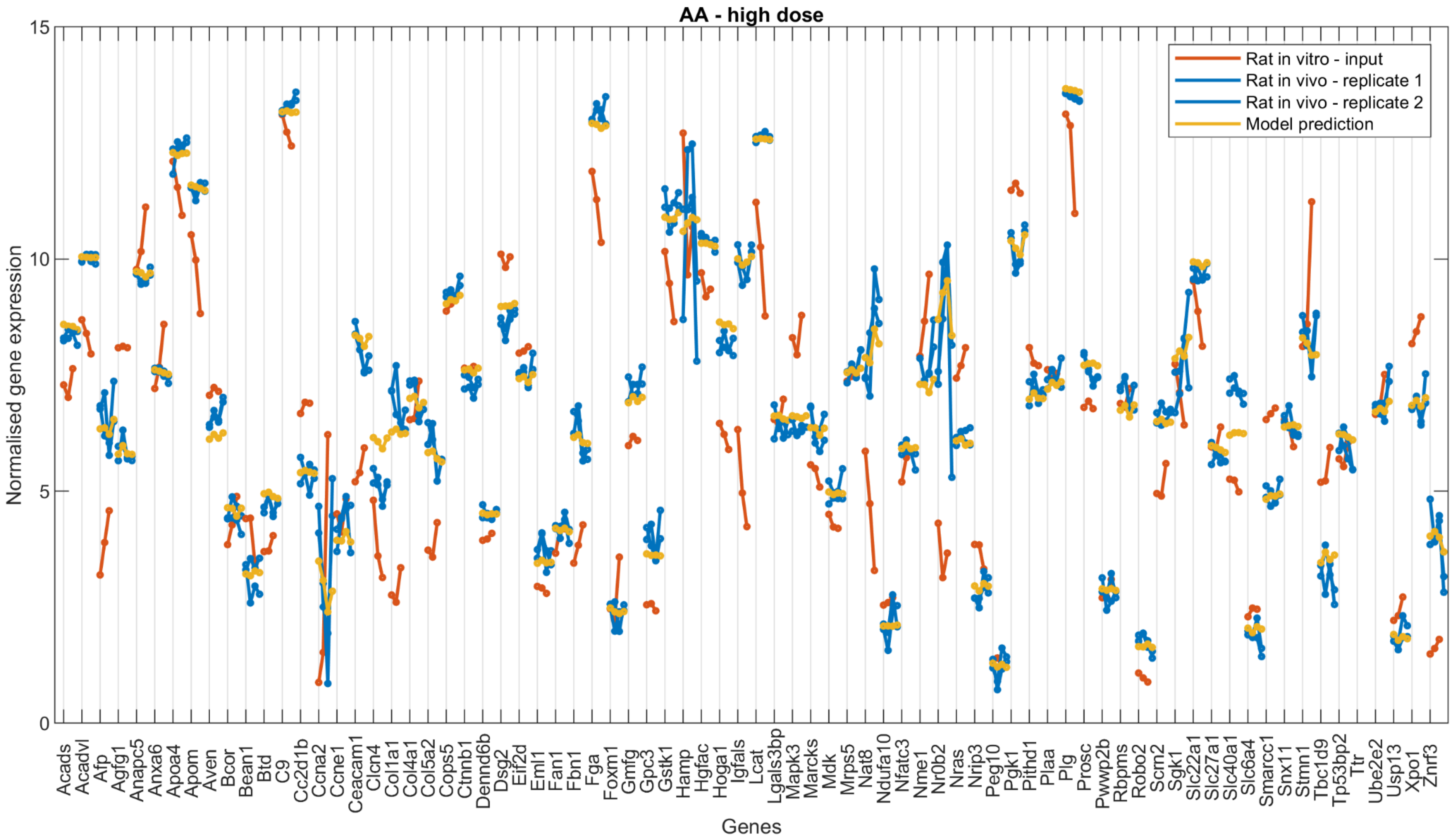

Figure S5c : Measured and model predicted time series of rat *in vitro* to rat *in vivo* gene expression for the full GTX/C gene set for the validation compound azathioprine (high dose) for the CNN model.

Measured time series of rat *in vitro* gene expression (red) following an exposure to a high dose of the validation compound azathioprine is input to the CNN model. The model predictions of rat *in vivo* gene expression are in yellow. Both biological replicates for the time series of rat *in vivo* gene expression following low dose exposure of azathioprine are depicted in blue for reference. Data is shown for the full GTX/C gene set (76 genes).

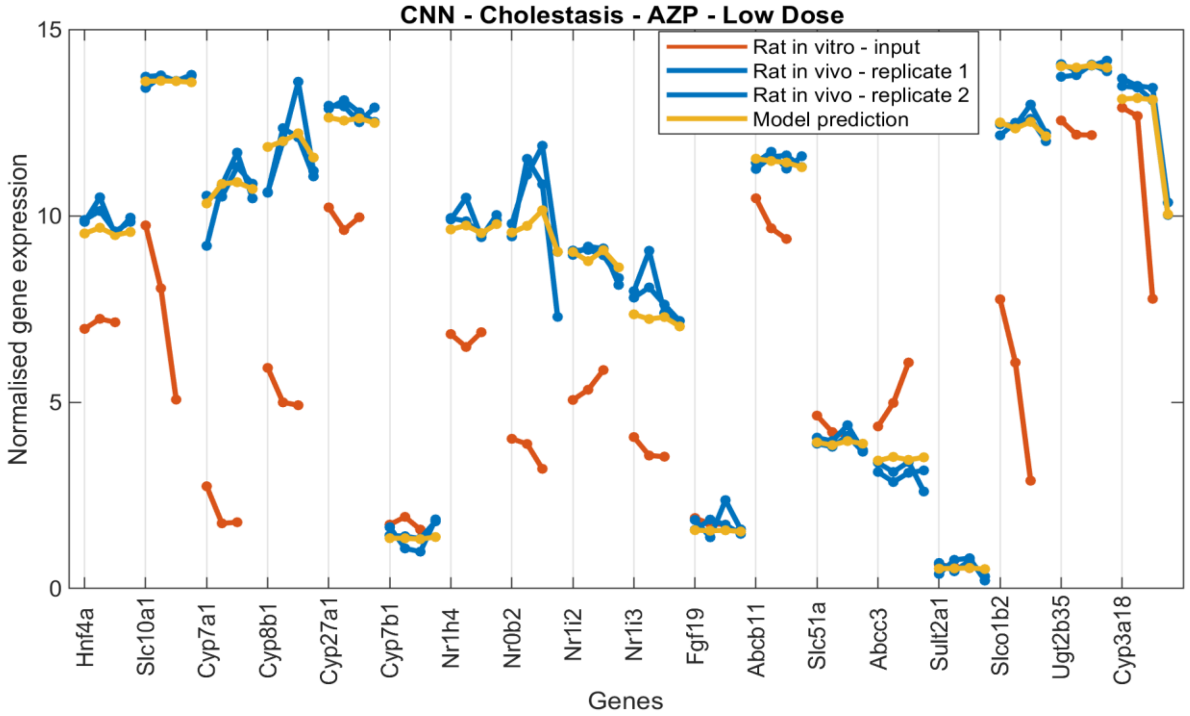

Figure S6a : Measured and model predicted time series of rat *in vitro* to rat *in vivo* gene expression for the Cholestasis gene set for the validation compound azathioprine (low dose) for the CNN model.

Measured time series of rat *in vitro* gene expression (red) following an exposure to a low dose of the validation compound azathioprine is input to the CNN model. The model predictions of rat *in vivo* gene expression are in yellow. Both biological replicates for the time series of rat *in vivo* gene expression following low dose exposure of azathioprine are depicted in blue for reference. Data is shown for the Cholestasis gene set (18 genes).

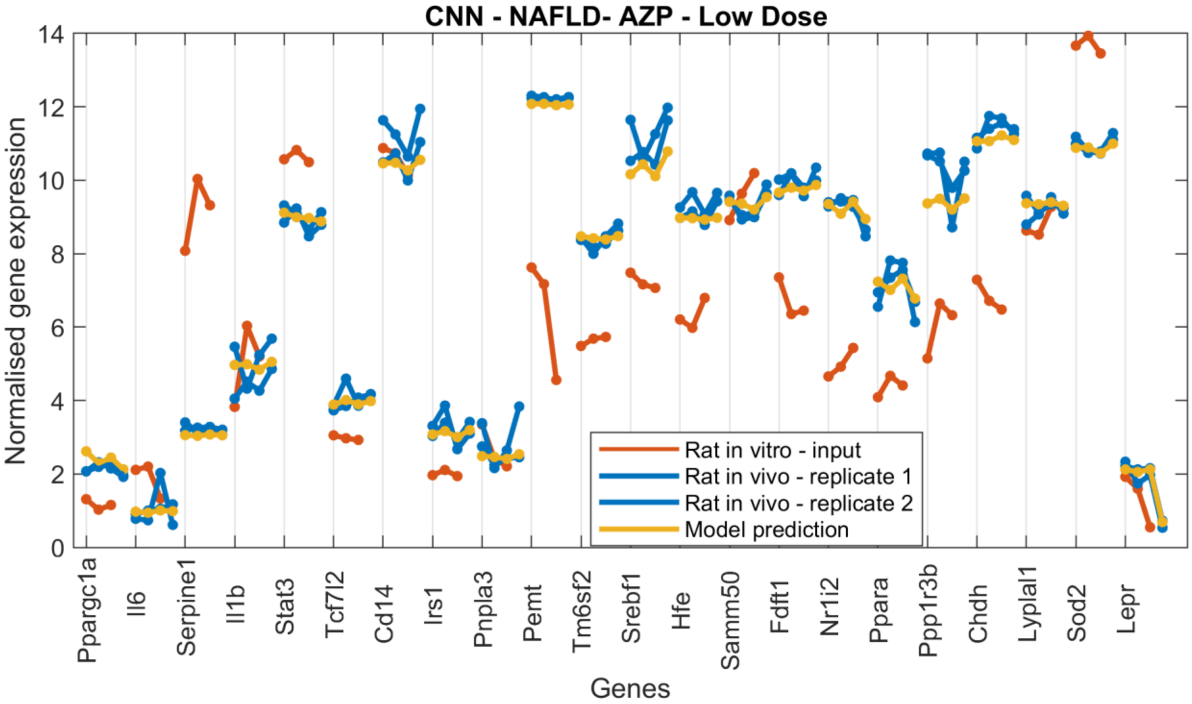

Figure S6b : Measured and model predicted time series of rat *in vitro* to rat *in vivo* gene expression for the NAFLD gene set for the validation compound azathioprine (low dose) for the CNN model.

Measured time series of rat *in vitro* gene expression (red) following an exposure to a low dose of the validation compound azathioprine is input to the CNN model. The model predictions of rat *in vivo* gene expression are in yellow. Both biological replicates for the time series of rat *in vivo* gene expression following low dose exposure of azathioprine are depicted in blue for reference. Data is shown for the NAFLD gene set (22 genes).

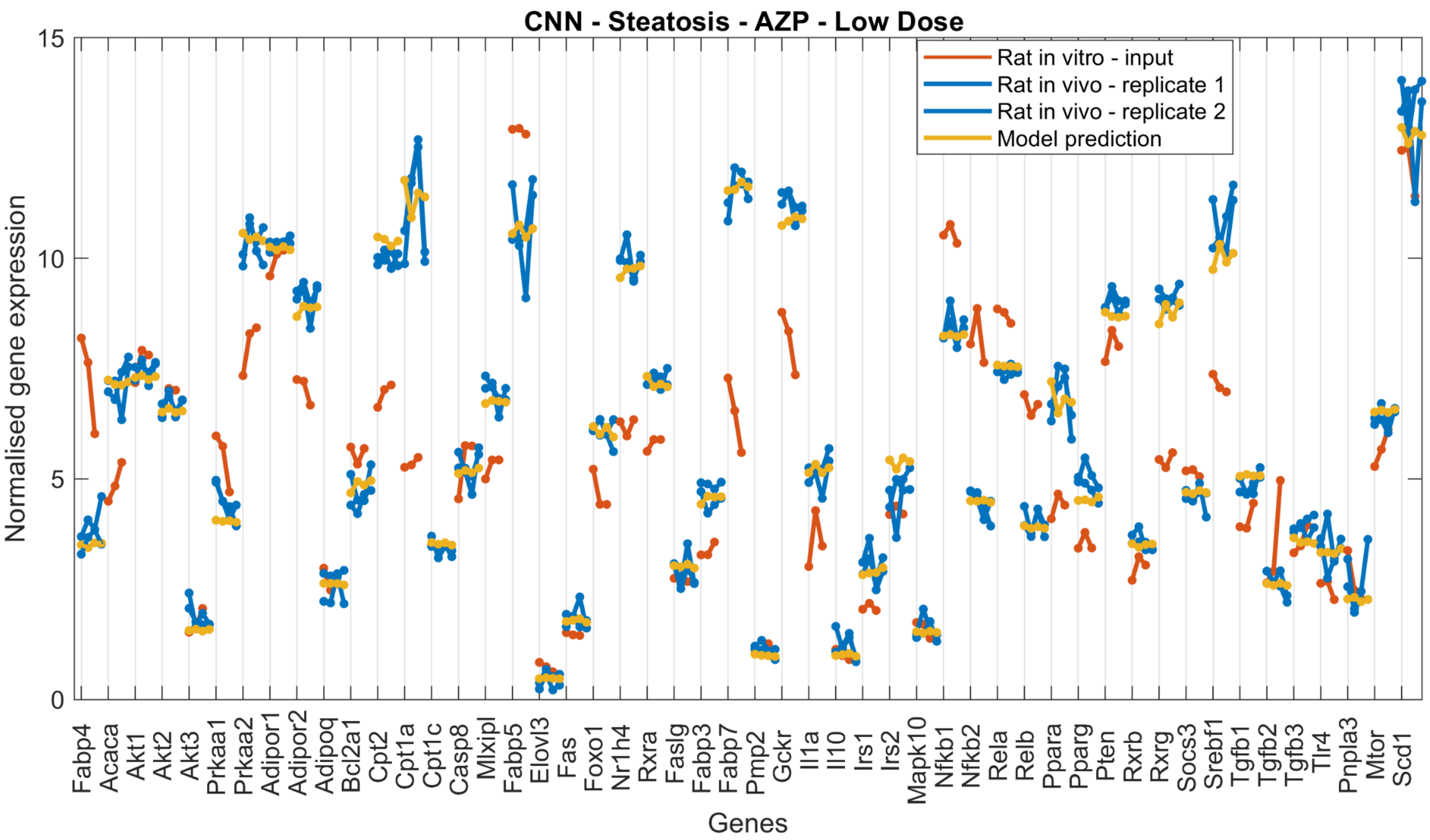

Figure S6c : Measured and model predicted time series of rat *in vitro* to rat *in vivo* gene expression for the Steatosis gene set for the validation compound azathioprine (low dose) for the CNN model.

Measured time series of rat *in vitro* gene expression (red) following an exposure to a low dose of the validation compound azathioprine is input to the CNN model. The model predictions of rat *in vivo* gene expression are in yellow. Both biological replicates for the time series of rat *in vivo* gene expression following low dose exposure of azathioprine are depicted in blue for reference. Data is shown for the Steatosis gene set (50 genes).

Supplementary material Section 2: additional analyses

<u>Nested sets of non-orthologus genes – rat *in vitro* to rat *in vivo.*</u>

As with the human *in vitro* predictions, as the models are trained on larger gene sets the average mean absolute error decreases for all models. The average mean absolute error values are greater for the rat *in vivo* predictions than for the human *in vitro* predictions. Higher average mean absolute error values for the rat *in vivo* predictions were also observed for the toxicologically relevant gene sets identified from literature.

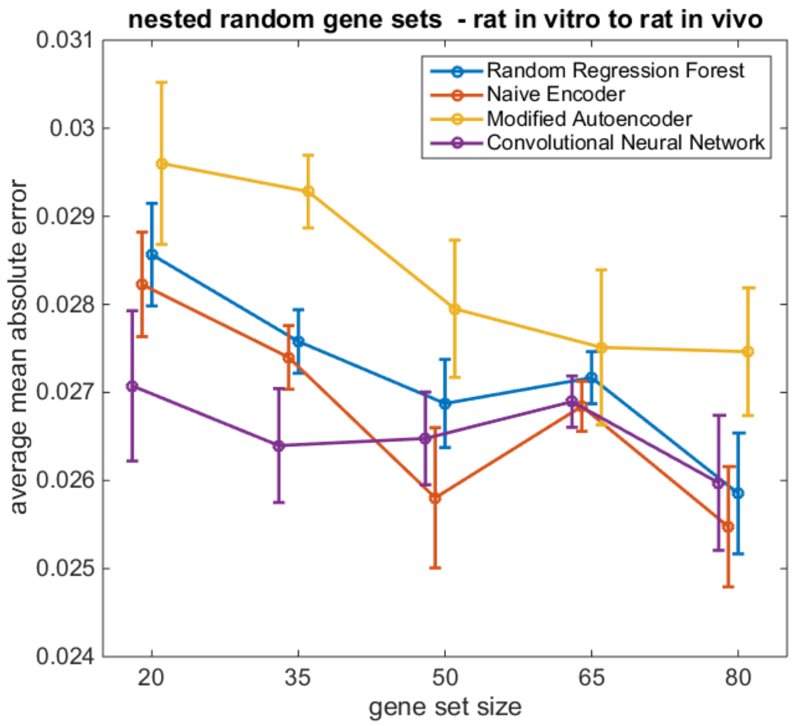

Figure S6:Average mean absolute error in predicted rat *in vivo* gene expression for each model trained on several nested sets of randomly selected genes of increasing size.

Each model included in the analysis (CNN, naïve encoder, modified autoencoder, and random regression forest) were trained to predict rat *in vivo* gene expression from rat *in vitro* gene expression on a population of randomly selected nested gene sets of increasing size (20, 35, 50, 60, 80 genes). The figure depicts the mean average mean absolute error for each model trained on a population of ten randomly generated non-orthologous gene sets of each size. The error bars indicate the standard error of the mean.

<u>Nested sets of known rat-human orthologs – rat *in vitro* to human *in vitro.*</u>

Orthologs are two, or more, homologous gene sequences found in different species related by linear decent. Ortholgs are commonly utilized to relate results from rodent *in vivo* and *in vitro* bio assays to the human system. In order to evaluate any added benefit of using known orthologs when predicting times series of human in vitro gene expression given a time series gene expression the models were also applied to randomly selected nested gene sets of increasing size of known rat human orthologs. The range of average mean absolute error values for predicting human in vitro gene expression from rat in vitro gene expression using known rat-human orthologs(Figure S6 below) shows no improvement over using randomly generated non-orthologous gene sets (Figure 8 in the text) This indicates there in no advantage in restricting gene predictions to known rat-human orthologs. This is unsurprising as all three deep learning models implemented in this system contain a bottleneck in their architecture. As a result, model predictions of a human gene expression pattern are made by a non-linear combination of the input rat gene expression patterns. As with the randomly selected non-orthologous gene sets, as more genes are included the average mean absolute error decreases for all models. Again, the convolutional neural network consistently out preforms the random regression forest, our benchmarking classical machine learning method.

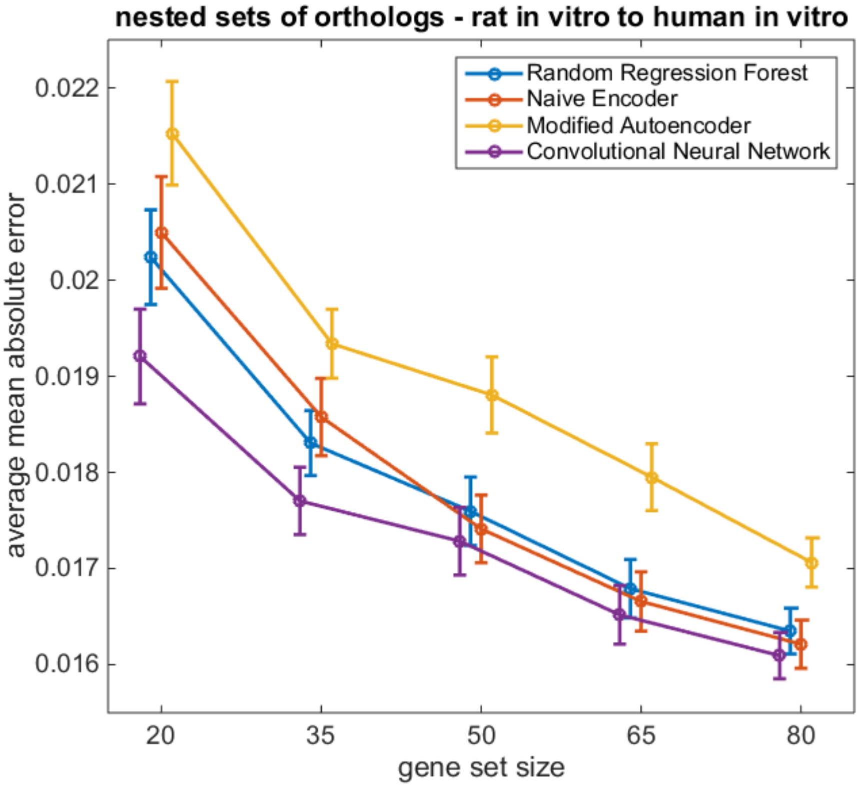

Figure S7 : Average mean absolute error in predicted human *in vitro* gene expression for each model trained on several nested sets of randomly selected rat-human orthologs of increasing size.

Each model included in the analysis (CNN, naïve encoder, modified autoencoder, and random regression forest) were trained to predict human *in vitro* gene expression from rat *in vitro* gene expression on a population of randomly selected nested gene sets of increasing size (20, 35, 50, 60, 80 genes). The figure depicts the mean average mean absolute error for each model trained on a population of thirty randomly generated gene sets of known rat-human orthologs of each size. The error bars indicate the standard error of the mean.

Supplementary material section 3 – Data information

Table 1 : Subset of compounds from TG-GATEs included in this analysis [1].

compound	abbreviation	Carcinogenicity	Genotoxicity
Acetaminophen	APAP	C	GTX
Adapin	ADP	unknown	unknown
Allopurinol	APL	unknown	GTX
Allyl alcohol	AA	unknown	unknown
Aspirin	ASA	NC	NGTX
Azathioprine	AZP	C	GTX
Benzbromarone	BBr	unknown	unknown
Bromobenzene	BBZ	unknown	NGTX
Carbamazepine	CBZ	unknown	unknown
Chlorpromazine	CPZ	unknown	NGTX
Cimetidine	CIM	NC	unknown
Clofibrate	CFB	C	GTX(*in vitro*), NGTX(*in vivo*)
Coumarin	CMA	C	GTX(*in vitro*), NGTX(*in vivo*)
Cyclophosphamide	CPA	C	GTX
Diazepam	DZP	NC	GTX
Diclofenac	DFNa	NC	NGTX
Ethionine	ET	unknown	unknown
Fluphenazine	FP	unknown	GTX
Flutamide	FT	unknown	unknown
Gemfibrozil	GFZ	unknown	unknown
Glibenclamide	GBC	unknown	unknown
Griseofulvin	GF	C	GTX(*in vitro*), NGTX(*in vivo*)
Haloperidol	HPL	unknown	NGTX
Hexachlorobenzene	HCB	C	GTX
Indomethacin	IM	unknown	unknown
Isoniazid	INAH	C	GTX
Ketoconazole	KC	unknown	unknown
Labetalol	LBT	unknown	NGTX
Lomustine	LS	C	GTX
Methapyrilene	MP	unknown	GTX
Methyltestosterone	MTS	unknown	unknown
Naphthyl isothiocyanate	ANIT	unknown	NGTX
Nitrofurantoin	NFT	C	GTX(*in vitro*), NGTX(*in vivo*)
Omeprazole	OPZ	unknown	NGTX
Perhexiline	PH	unknown	unknown

Phenobarbital	PB	C	GTX(*in vitro*), NGTX(*in vivo*)
Phenylbutazone	PhB	C	GTX(*in vitro*), NGTX(*in vivo*)
Phenytoin	PHE	C	NGTX
Propylthiouracil	PTU	C	NGTX
Rifampicin	RIF	unknown	unknown
Sulfasalazine	SS	C	NGTX
Tetracycline	TC	C	GTX
Thioacetamide	TAA	C	GTX
Thioridazine	TRZ	unknown	unknown
Valproic acid	VPA	unknown	NGTX
WY-14643	WY	C	NGTX

Overview of gene sets included in these analyses

Table 2: Cholestasis gene set identified from literature [2-4]

HNF4A	SLC10A1	SLCO1B1	CYP7A1	CYP8B1
CYP27A1	CYP7B1	NR1H4	NR0B2	N21L2
NR1L3	FGF19	ABCB11	SLC51A	ABCC3
	UGT2B4	CYP3A4	SULT2A1	

Table 3: NAFLD gene set identified from literature [5].

PPARGC1A	IL6	SERPIN1	IL1B	STAT3	TCF7L2
PNPLA3	PEMT	TM6SF2	SREBF1	HFE	SAMM50
FDFT1	NR1I2	PPARA	PPP1R3B	CHDH	LYPLAL1
		SOD2	LEPR		

Table 4: Steatosis gene set; generated in house.

FABP4	ACACA	AKT1	AKT2	AKT3	PRKAA1	PRKAA2	ADIPOR1
ADIPOR2	ADIPOQ	BCL2A1	CPT2	CPT1A	CPT1C	CASP8	MLXIPL
FABP5	ELOVL3	FAS	FOXO1	NR1H4	RXRA	FASLG	FABP3
FABP7	PMP2	GCKR	IL1A	IL10	IRS1	IRS2	MAPK10
NFKB1	NFKB2	RELA	RELB	PPARA	PPARG	PTEN	RXRB
RXRG	SCD	SOCS3	SREBF1	TGFB1	TGFB2	TGFB3	TLR4
		MTOR			PNPLA3		

The steatosis gene set was generated in house combining a literature search using the search terms “liver steatosis”, “Nonalcoholic fatty liver disease”, and “NAFLD”, the steatosis pathway from KEGG (hsa04932) and the steatosis adverse outcome pathway from Wikipathways. In addition genes were filtered to include only known human –rat orthologs measured by both the Rat Genome 230 2.0 Array and the Human Genome U133 Plus 2.0 Array used in this study. This gave rise to a seed gene set of 45 genes, these were then used an input for input for MetaCore (version 6.30, build 68780, accessed on 9th of May 2017) to generate a fully connected gene interaction network. Dijkstra's shortest path algorithm is used to construct the network with allowing one node to be added if necessary. This analysis yielded the final set of 50 genes.

Table 5: gene set reported as being a genomic signature of genotoxicity/carcinogenicity [6,7].

CEACAM1	CLCN4	EML1	PWWP2B	UBE2E2	USP13	GMFG
PROSC	TTR	NR0B2	NAT8	RBPMS	TBC1D9	SNX11
BCOR	ROBO2	DENND6B	APOM	NR1P3	PITHD1	AVEN
ZNRF3	BEAN1	SLC27A1	ANXA6	APOA4	BTD	EIF2D
AGFG1	NDUFA1-	NFATC3	PLAA	FAN1	SLC40A1	ANAPC5
MRPS5	GSTK1	HOGA1	FGA	SGK1	SLC6A4	SCRN2
CC2D1B	GPC3	MDK	COL5A2	TP53BP2	XPO1	AFP
CCNA2	CCNE1	COL1A1	COL4A1	CTNNB1	FBN1	FOXM1
STMN1	LGALS3BP	MARCKS	NME1	NRAS	PGK1	MAPK3
SMARCC1	COPS5	PEG10	HGFAC	IGFALS	LCAT	SLC22A1
ACADS	ACADVL	C9	DSG2	PLG	HAMP	

References

1. Igarashi Y, Nakatsu N, Yamashita T, et al. Open TG-GATEs: a large-scale toxicogenomics database. *Nucleic Acids Res*. 2014;43(Database issue):D921-7.

2. Jung, D., Elferink, M. G. L., Stellaard, F., Groothuis, G. M. M.. "Analysis of bile acid-induced regulation of FXR target genes in human liver slices," *Liver Int.*, 27(1), 137–144 (2007).

3. Vinken, M., Landesmann, B., Goumenou, M.,Vinken, S., Shah, I., Jaeschke, H. et al. "Development of an Adverse Outcome Pathway From Drug-Mediated Bile Salt Export Pump Inhibition to Cholestatic Liver Injury," *Toxicol. Sci.*, 136,. 97–106, (2013).

4. Liu, J., Lu, H.. Lu, Y.-F., Lei, X., Cui,. Ellis, J. Y E et al. "Potency of individual bile acids to regulate bile acid synthesis and transport genes in primary human hepatocyte cultures.," *Toxicol. Sci.*, 141, 538–46 (2014).

5. Ryaboshapinka M, Hammar M. "Human hepatic gene expression signature of non-alcoholic fatty liver disease progression, a meta-analysis." *Scientific Reports* 7:12361(2017)

6. Magkoufopoulou, C., Claessen, S.M.H., Tsamou, M., Jennen, D.G.J., Kleinjans, J.C.S., van Delft; J.H.M. "A transcriptomics-based *in vitro* assay for predicting chemical genotoxicity *in vivo",Carcinogenesis*, 33, 1421–1429,(2012)

7. Caiment, F., Tsamou, M., Jennen, D., Kleinjans J. Assessing compound carcinogenicity *in vitro* using connectivity mapping, *Carcinogenesis,* 35, 201–207, (2014)
